# Exploring the missing heritability in subjects with hearing loss, enlarged vestibular aqueducts, and a single or no pathogenic *SLC26A4* variant

**DOI:** 10.1007/s00439-021-02336-6

**Published:** 2021-08-19

**Authors:** Jeroen J. Smits, Suzanne E. de Bruijn, Cornelis P. Lanting, Jaap Oostrik, Luke O’Gorman, Tuomo Mantere, DOOFNL Consortium, M. F. van Dooren, S. G. Kant, H. H. W. de Gier, E. H. Hoefsloot, M. P. van der Schroeff, L. J. C. Rotteveel, F. G. Ropers, J. C. C. Widdershoven, J. R. Hof, E. K. Vanhoutte, I. Feenstra, H. Kremer, C. P. Lanting, R. J. E. Pennings, H. G. Yntema, R. H. Free, J. S. Klein Wassink-Ruiter, R. J. Stokroos, A. L. Smit, M. J. van den Boogaard, F. A. Ebbens, S. M. Maas, A. Plomp, T. P. M. Goderie, P. Merkus, J. van de Kamp, Frans P. M. Cremers, Susanne Roosing, Helger G. Yntema, Erik de Vrieze, Ronny Derks, Alexander Hoischen, Sjoert A. H. Pegge, Kornelia Neveling, Ronald J. E. Pennings, Hannie Kremer

**Affiliations:** 1 Hearing and Genes, Department of Otorhinolaryngology, Radboud University Medical Center, Nijmegen, The Netherlands; 2 Department of Human Genetics, Radboud University Medical Center, Internal Postal Code 855, P.O. Box 9101, 6500 HB Nijmegen, The Netherlands; 3 Donders Institute for Brain, Cognition and Behaviour, Radboud University Medical Center, Nijmegen, The Netherlands; 4 Laboratory of Cancer Genetics and Tumor Biology, Cancer and Translational Medicine Research Unit and Biocenter Oulu, University of Oulu, Oulu, Finland; 5 Center for Infectious Diseases (RCI), Department of Internal Medicine, Radboud University Medical Center, Nijmegen, The Netherlands; 6 Radboud Institute for Molecular Life Sciences, Radboud University Medical Center, Nijmegen, The Netherlands; 7 Radboud Expertise Center for Immunodeficiency and Autoinflammation and Center for Infectious Disease (RCI), Radboud University Medical Center, Nijmegen, The Netherlands; 8 Department of Radiology and Nuclear Medicine, Radboud University Medical Center, Nijmegen, The Netherlands

## Abstract

Pathogenic variants in *SLC26A4* have been associated with autosomal recessive hearing loss (arHL) and a unilateral or bilateral enlarged vestibular aqueduct (EVA). *SLC26A4* is the second most frequently mutated gene in arHL. Despite the strong genotype–phenotype correlation, a significant part of cases remains genetically unresolved. In this study, we investigated a cohort of 28 Dutch index cases diagnosed with HL in combination with an EVA but without (M0) or with a single (M1) pathogenic variant in *SLC26A4*. To explore the missing heritability, we first determined the presence of the previously described EVA-associated haplotype (Caucasian EVA (CEVA)), characterized by 12 single nucleotide variants located upstream of *SLC26A4*. We found this haplotype and a delimited V1-CEVA haplotype to be significantly enriched in our M1 patient cohort (10/16 cases). The CEVA haplotype was also present in two M0 cases (2/12). Short- and long-read whole genome sequencing and optical genome mapping could not prioritize any of the variants present within the CEVA haplotype as the likely pathogenic defect. Short-read whole-genome sequencing of the six M1 cases without this haplotype and the two M0/CEVA cases only revealed previously overlooked or misinterpreted splice-altering *SLC26A4* variants in two cases, who are now genetically explained. No deep-intronic or structural variants were identified in any of the M1 subjects. With this study, we have provided important insights that will pave the way for elucidating the missing heritability in M0 and M1 *SLC26A4* cases. For pinpointing the pathogenic effect of the CEVA haplotype, additional analyses are required addressing defect(s) at the RNA, protein, or epigenetic level.

**Supplementary Information:**

The online version contains supplementary material available at 10.1007/s00439-021-02336-6.

## Introduction

*SLC26A4* encodes the transmembrane anion transporter pendrin and is most abundantly expressed in the inner ear, thyroid gland, kidney, and airways epithelia (Everett et al. 1997, 1999; Pedemonte et al. 2007; Royaux et al. 2000, 2001). The 780 amino acid protein is part of the solute carrier family 26 and plays a pivotal role in chloride, bicarbonate and iodine transport. In the inner ear, pendrin functions as a Cl^−^/HCO_3_^−^ exchanger. The protein is expressed in the epithelial cells of the cochlea (outer sulcus and spindle cells), the vestibular labyrinth (transitional cells), and the endolymphatic duct and sac (mitochondrial-rich cells) (Wangemann 2011; Wangemann et al. 2007). Expression of pendrin is essential for the development of the (murine) auditory and vestibular system and for maintaining ion homeostasis in the endolymphatic fluid and the endocochlear potential (Dou et al. 2004; Everett et al. 1999; Royaux et al. 2003; Wangemann 2011).

Defects in *SLC26A4* are among the most frequent causes (up to 10%) of early-onset autosomal recessive hearing loss (arHL); non-syndromic DFNB4 (MIM: 600,791) and Pendred syndrome (MIM: 274,600) (Sloan-Heggen et al. 2016). Individuals carrying biallelic pathogenic *SLC26A4* variants are affected by variable, often progressive and predominantly sensorineural HL with a congenital or childhood-onset (Lee et al. 2014; Suzuki et al. 2007). In Pendred syndrome, the HL phenotype is accompanied by an iodine organification defect that can lead to thyroid goiter (Fraser 1965). In individuals affected by either syndromic or non-syndromic *SLC26A4-*associated HL, a unilateral or bilateral enlarged vestibular aqueduct (EVA) is observed, which is the most common imaging abnormality in individuals with HL (van Beeck Calkoen et al. 2017, 2018). In some cases, EVA can be part of Mondini dysplasia: an inner ear malformation that includes both EVA and cochlear incomplete partition type II. Although Mondini dysplasia can be observed in both Pendred syndrome and DFNB4 cases, cases with the syndromic type of HL are more likely to present Mondini dysplasia than those with non-syndromic HL (Forli et al. 2021; Mey et al. 2019).

Pathogenic variants in *SLC26A4* have a loss-of-function effect, leading to malfunctioning of the pendrin ion transporter. Besides the antenatal formation of an EVA, this ultimately leads to acidification of the endolymphatic fluids in the inner ear during embryonic development (Griffith and Wangemann 2011; Wangemann 2011). Although the exact molecular pathogenic mechanism remains to be elucidated, the lack of pendrin function ultimately leads to degeneration of the sensory cells in the inner ear (Wangemann 2011).

Despite the strong association between defects of *SLC26A4* and HL combined with an EVA, genetic screening of subjects with this combination of defects often does not reveal biallelic pathogenic variants in *SLC26A4* (coined M2). Cohort studies report that 14–31% of the subjects with an EVA and HL carry a monoallelic pathogenic variant in *SLC26A4* (M1), whereas in 10–65% of the subjects, no potentially pathogenic variant in the coding or splice site regions of the gene can be identified (M0) (Azaiez et al. 2007; Choi et al. 2009; Mey et al. 2019). Segregation analyses performed in family members of M1 subjects, however, do suggest that in 98% of M1 subjects an unidentified or unrecognized variant is present on the *trans SLC26A4* allele (Azaiez et al. 2007; Pryor et al. 2005). In line with this hypothesis, Chattaraj and coworkers reported a haplotype, referred to as the Caucasian EVA (CEVA) haplotype, that was present in 13 of 16 (81%) of the studied M1 families and that was also enriched in M0 subjects (Chattaraj et al. 2017). The haplotype is defined by the combination of 12 single nucleotide polymorphisms (SNPs; allele frequency (AF) 1.9–4.0%) spanning a 613 kb region. The 12 SNPs are located within a region of linkage disequilibrium that extends from upstream of *PRKAR2B* to intron 3 of *SLC26A4* and are either intergenic or intronic of the genes *SLC26A4*, BCAP29, *DUS4L, COG5*, *GPR22*, *HBP1*, *PRKAR2B* and *PIK3CG* (Chattaraj et al. 2017). The true genetic defect of the CEVA allele has not been identified yet, but it cannot be excluded that a potential defect was missed due to the technical limitations of short-read sequencing and other standard-of-care tests. The CEVA haplotype was reported to be associated with a less severe HL phenotype as compared to variants in the protein-coding or splice site regions of *SLC26A4* (Chao et al. 2019).

We investigated a Dutch cohort of M1 and M0 subjects with HL and a unilateral or bilateral EVA. All subjects were tested for the presence of the CEVA haplotype, and whole-genome sequencing (WGS) was performed to detect potentially missed single nucleotide variants (SNVs), structural variants (SVs), and regulatory or deep-intronic variants. Long-read sequencing and optical genome mapping were performed to reveal a potentially missed SV located on the CEVA haplotype. Variants located within the haplotype were subjected to in silico analyses to investigate potential effects on the regulation of *SLC26A4* expression or on splicing. With this study, we provided further insights into *SLC26A4*-associated disease.

## Material and methods

### Inclusion criteria and clinical evaluation

Subjects diagnosed with unilateral or bilateral HL and a unilateral or bilateral EVA on CT or MRI and for whom medical genetic testing only revealed a heterozygous (M1, *n* = 16) or no pathogenic variant (M0, *n* = 12) in *SLC26A4* were eligible to participate in this study. A retrospective cohort of nine subjects with confirmed pathogenic (biallelic) variants in *SLC26A4* was added as a reference cohort (Online Resource Table S1).

Medical history was taken from all participants and special attention was paid to non-genetic causes of HL. Results of pure tone, speech, and brainstem evoked response audiometry, performed in a sound-attenuated booth, were collected. Air and bone conduction pure tone thresholds were determined for frequencies ranging from 0.25 to 8 kHz. Threshold estimates based on brainstem evoked response audiometry were used when pure tone audiometry was not available. Individuals were considered affected when pure tone thresholds for at least three frequencies were above the frequency-specific 95^th^ percentile of age- and sex-specific thresholds (ISO 7029:2017) for the best hearing ear. In the Netherlands, routine newborn hearing screening is carried out by the detection of transient evoked otoacoustic emissions (van der Ploeg et al. 2012). When available, these data were used to determine whether the HL was congenital.

Previously performed CT and MRI scans were retrieved and reassessed by an experienced neuroradiologist (SAHP). An EVA was defined as a vestibular aqueduct that measured ≥ 2 mm at the operculum and/or ≥ 1 mm at the midpoint (Boston et al. 2007), in accordance with previously published reports on this topic (Chao et al. 2019; Chattaraj et al. 2017). Analyses of pair-wise differences between patient groups were performed with R (R Foundation, Auckland, New Zealand) using multivariate linear regression analysis (using lsmeans 2.3.0) with a correction for multiple comparisons using the Holm method (Lenth 2016).

### Next-generation sequencing and variant interpretation

Genomic DNA was isolated from peripheral blood lymphocytes and analyzed by molecular inversion probe (MIP) sequencing, whole-exome sequencing (WES) or whole-genome sequencing (WGS) (Online Resource Table S2). For WES, exome enrichment was performed using the Agilent SureSelect Human All Exome V4 or V5 kits according to the manufacturer’s instructions. Subsequently, sequencing was executed on an Illumina HiSeq system by BGI Europe (Copenhagen, Denmark), with a minimal coverage of 20× for 93.77% of the targets and an average coverage of > 100 reads. Read mapping along the hg19 reference genome (GRCh37/hg19) and variant calling was performed using BWA V.0.78 (Li and Durbin 2009) and GATK HaplotypeCaller V.3.3 (McKenna et al. 2010), respectively. An in-house developed pipeline was used for variant annotation and copy number variant (CNV) detection was performed using CoNIFER V.0.2.2.3 (Krumm et al. 2012). WGS was performed by BGI (Hongkong, China) on a BGISeq500 using a 2 × 100 bp paired-end module, with a minimal median coverage of 30-fold per genome. Read mapping (GRCh37/hg19) and variant calling was performed as described for WES. Structural variants (SVs) were called using the Manta Structural Variant Caller V.1.1.0 (SV detection based on paired end and split read evidence) (Chen et al. 2016) and CNVs using Control-FREEC (CNV detection based on alterations in read depth) (Boeva et al. 2012). MIP design, sequencing and data analysis were performed as previously described (de Bruijn et al. 2021; Neveling et al. 2017). MIPs were designed to cover exons and exon–intron boundaries of a panel of 120 HL genes (Online Resource Table S3). For each targeted region an average coverage of > 500 reads was obtained. A minimal coverage of 20× was reached for 91.78% of the MIPs. CNV detection for *SLC26A4* was performed using a read coverage analysis as previously described (Khan et al. 2020). Additionally, coding and splice site regions of *FOXI1* and the regions harboring reported pathogenic variants in *EPHA2* were sequenced using Sanger sequencing as previously described (Wesdorp et al. 2018), since these genes are not included in the MIP panel. Primer sequences and PCR conditions are available upon request.

Variant prioritization was based on an AF of ≤ 0.5% [gnomAD V2.1.1 (Karczewski et al. 2019) and our in-house exome database (~ 15,000 alleles)], unless specified otherwise. Variant visualization was performed using the IGV software V.2.4 (Broad Institute, Cambridge, MA, USA) (Robinson et al. 2011). Interpretation of missense variants was performed using the in silico tools CADD-PHRED (≥ 15) (Kircher et al. 2014), SIFT (≤ 0.05) (Vaser et al. 2015), PolyPhen-2 (≥ 0.450) (Adzhubei et al. 2010) and MutationTaster (deleterious) (Schwarz et al. 2014) to predict potentially deleterious effects. Variants were prioritized if a deleterious effect was predicted by at least two of these tools. Candidate variants were validated by Sanger sequencing and segregation analysis was performed when DNA of family members was available. Primer sequences and PCR conditions are available upon request. Potential effects on splicing of missense, synonymous and intronic variants were assessed using the deep-learning splice prediction algorithm SpliceAI (≥ 0.1) (Jaganathan et al. 2019). The maximum distance between the variant and potential gained or lost splice sites was set to 1000 bp. Predicted splice altering defects were evaluated using an in vitro splice assay in HEK293T cells as previously described (Sangermano et al. 2018).

### Detection of the CEVA haplotype

Initial identification of the CEVA haplotype was performed with SNP-genotyping by Sanger sequencing in index cases for whom parental DNA was available for segregation analysis. Subsequently, the corresponding VNTR marker haplotype was determined in CEVA-positive families. For additional cases, VNTR marker analysis was performed to enable a fast and cost-effective detection of the CEVA haplotype. For the VNTR marker analysis, DNA segments were amplified by employing touchdown PCR, and subsequent analysis was carried out on an ABI Prism 3730 Genetic Analyzer (Applied Biosystems, Foster City, CA, USA). Genomic positions of the markers were determined using the UCSC genome browser (GRCh37/hg19) (Kent et al. 2002). Alleles were assigned with the GeneMarker software (V.2.6.7, SoftGenetics, State College, PA, USA) according to the manufacturer’s instructions. When an individual was suspected of carrying the CEVA haplotype based on VNTR-marker alleles, SNP genotyping by Sanger sequencing was performed to confirm the presence of the twelve SNPs that are located within the haplotype (Chattaraj et al. 2017). SNP-phasing was performed if DNA samples of family members was available.

### Optical genome mapping

Optical genome mapping (Bionano Genomics, San Diego, CA, USA) was performed as previously described (Mantere et al. 2021; Neveling et al. 2021). Ultra-high molecular weight DNA was isolated from whole peripheral blood (collected in EDTA tubes) using the SP Blood & Cell Culture DNA Isolation Kit (Bionano Genomics, San Diego, CA, USA). CTTAAG labeling was performed using the DLS (Direct Label and Stain) DNA Labeling Kit (Bionano Genomics, San Diego, CA, USA) and the labeled sample was analyzed using a 3 × 1300 Gb Saphyr chip (G2.3) on a Saphyr instrument (Bionano Genomics, San Diego, CA, USA). An effective coverage of 124 × was reached, with a label density of 14.63/100 kb and an average N50 of 279 kb. De novo assembly (using GRCh37 and GRCh38) and variant annotation were performed using Bionano Solve version 3.4, which includes two separate algorithms for SV and CNV detection. Annotated variants were filtered for rare events as described previously (Mantere et al. 2021). In addition, the genomic region spanning the CEVA haplotype was analyzed visually in Bionano Access version 1.4.3.

### PacBio long-read sequencing

Genomic DNA was isolated from peripheral blood according to standard procedures and subjected to long-read genome Hi-Fi sequencing using the SMRT sequencing technology (Pacific Biosciences, Menlo Park, CA, USA). Library preparation was performed using the SMRTbell™ Template Prep Kit 2.0 (Pacific Biosciences, Menlo Park, CA, USA) following the manufacturer’s instructions. Size selection was performed using a BluePippin DNA size selection system (target fragments ~ 15–18 kb). Sequence primer V2 and polymerase 2.0 were used for binding. Subsequently, the SMRTbell library was loaded on an 8 M SMRTcell and sequencing was performed on a Sequel II system (Pacific Biosciences, Menlo Park, CA, USA). Circular consensus sequencing (CCS), Hi-Fi reads, were generated using the CCS (v4.2.0) tool and were aligned to the GRCh37/hg19 reference genome with pbmm2 (v.1.3.0). The unique molecular yield was 93.46 Gb and the post-alignment Hi-Fi- coverage was 12× [Mosdepth v0.3.1, (Pedersen and Quinlan 2018)]. SV calling was performed using PBSV (v2.4.0) and annotation was applied using an in-house SV annotation pipeline.

## Results

### Patient inclusion and genetic prescreening

In this study, we included 28 Dutch index cases diagnosed with a unilateral or bilateral EVA and unilateral or bilateral HL. All individuals were prescreened for pathogenic variants in *SLC26A4* (NM_000441.1) in a diagnostic setting and complete coverage of the coding and splice site (± 14 nucleotides) regions of *SLC26A4* was confirmed. In 16 individuals, a heterozygous (likely) pathogenic *SLC26A4* variant was reported and these cases were deemed M1. In the remaining 12 individuals, no potentially pathogenic variants were found in the coding or splice site regions of this gene, and these subjects were therefore considered M0. Causative variants in other genes associated with arHL (Van Camp and Smith 2021) were addressed and excluded by analyzing available sequencing data (WES or MIPs-based) or in WGS data obtained in this study (Online Resource Table S2). This revealed no homozygous or compound heterozygous variants that were known or predicted to be pathogenic, except two compound heterozygous variants in *OTOGL* (NM_173591.3) in individual SLC012 (Online Resource Table S4). The c.890C > T (p.(Pro297Leu)) variant in *OTOGL* has, however, been reported as (likely) benign in ClinVar (Landrum et al. 2018) and the Deafness Variation Database (Azaiez et al. 2018) and is classified as likely benign according to the ACMG guidelines (Oza et al. 2018). The c.1369G > T [p.(Val457Leu)] is considered as a variant of unknown significance (ACMG classification). Furthermore, subject SLC012 has progressive high-frequency HL, which differs from the symmetric, moderate, and stable HL associated with *OTOGL* (DFNB84B) (Oonk et al. 2014; Yariz et al. 2012). Therefore, we considered the identified *OTOGL* variants as non-causative. For none of the cases, (likely) pathogenic variants (UV4/UV5, ClinVar) were identified in genes associated with autosomal dominant HL or syndromic HL (Van Camp and Smith 2021).

### The CEVA haplotype is enriched in Dutch monoallelic *SLC26A4* cases

**Fig. 1:**
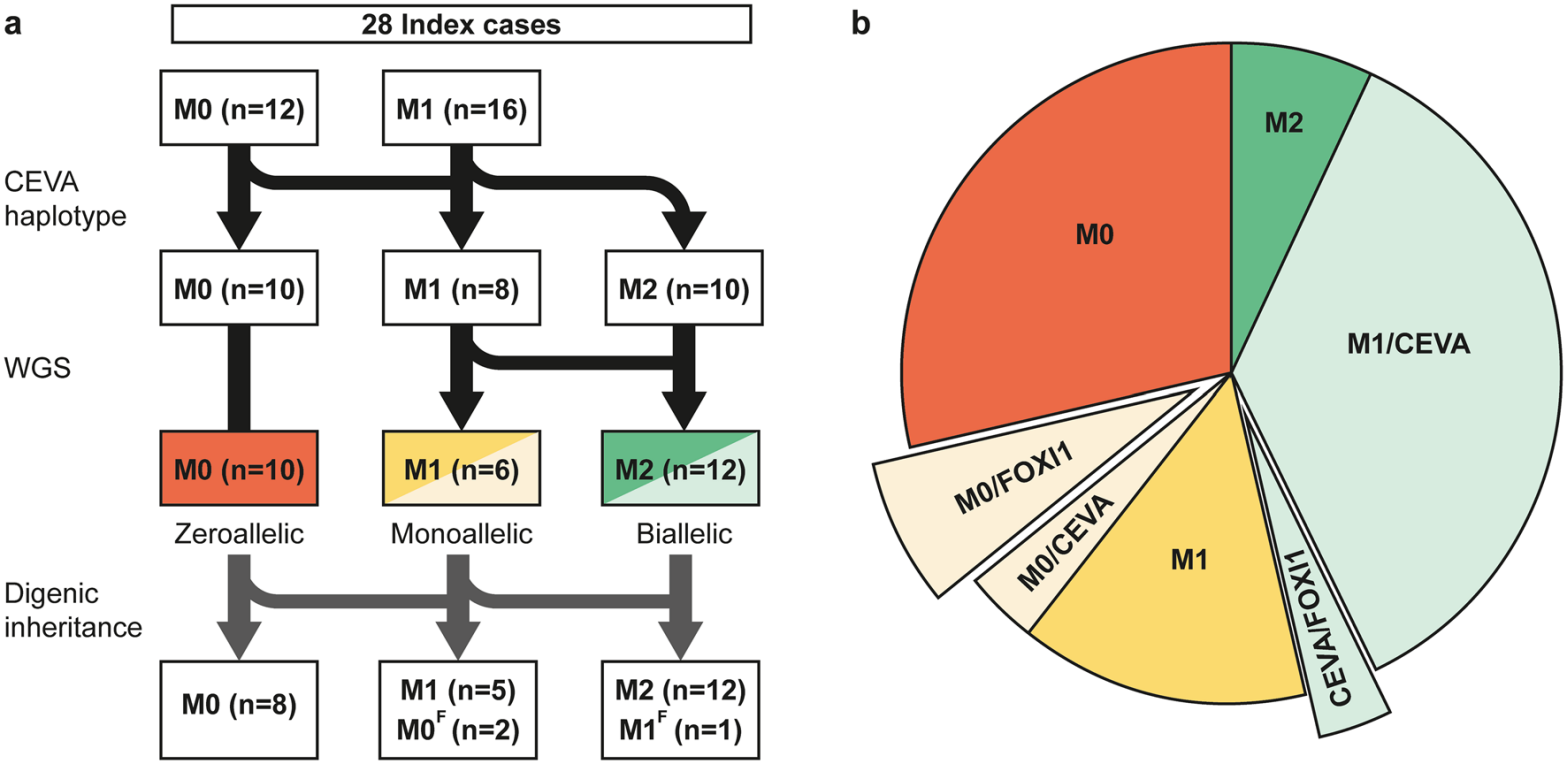
Overview of genetic analyses performed in zeroallelic and monoallelic *SLC26A4* cases*.* **a**, **b** To explain the missing heritability in zeroallelic (M0, *n* = 12) and monoallelic (M1, *n* = 16) *SLC26A4* cases, different genetic analyses were performed. First, individuals were screened for the presence of the CEVA haplotype (M0/CEVA, *n* = 2; M1/CEVA, *n *= 10). Second, whole-genome sequencing (WGS) was performed in all monoallelic cases (M0/CEVA, M1) to identify potential structural, splice (M2, *n* = 2) or regulatory variants. Finally, sequencing data were screened for potentially pathogenic variants in the *EPHA2*, *FOXI1* and *KCNJ10* genes. Digenic inheritance has been previously suggested for variants in these genes and the *SLC26A4* gene. In three cases (M0/FOXI1 (M0^F^), *n* = 2, CEVA/FOXI1 (M1^F^), *n* = 1), a potentially pathogenic variant in *FOXI1* (NM_012188.4, c.677C > T) was identified

In 2017, Chatteraj et al. described the ≥ 613-kb CEVA haplotype located centromeric of the *SLC26A4* gene to be enriched in M1 *SLC26A4* cases and M0 cases with HL and EVA (Chattaraj et al. 2017). To investigate whether this haplotype is also enriched in the selected Dutch cohort of M0 and M1 *SLC26A4* cases, we screened for the presence of this haplotype using VNTR marker analysis followed by Sanger sequencing of the 12 CEVA-associated SNPs. The CEVA haplotype was detected in 8 out of 16 (50%) M1 individuals and 2 out of 12 (16.7%) M0 subjects (Fig. 1, Table 1). In two additional M1 individuals (SLC040 and SLC071), only a partial CEVA haplotype was found, harboring 9/12 SNPs. We will refer to this smaller haplotype as the variant 1-CEVA (V1-CEVA) haplotype.

**Table 1:** Detection of the CEVA haplotype in M1 and M0 individuals

Case	Allele 1		Allele 2
	Variant	ACMG	CEVA
Zeroallelic *SLC26A4* cases			
SLC014	c.2059G > T; p.(Asp687Tyr)	UV3	
SLC015	–	–	
SLC017	–	–	
SLC039	–	–	ACACATG-GC-C (**CEVA**)
SLC043	–	–	
SLC052	–	–	
SLC069	–	–	
SLC070	–	–	
SLC073	–	–	
SLC080	–	–	ACACATG-GC-C (**CEVA**)
SLC084	–	–	
SLC086	–	–	
Monoallelic *SLC26A4* cases			
SLC002	c.412G > T; p.(Val138Phe)	UV5	
SLC003	c.131dup; p.(Thr45Aspfs*42)	UV5	ACACATG-GC-C (**CEVA**)
SLC012^a^	c.707 T > C; p(Leu236Pro)	UV5	ACACATG-GC-C (**CEVA**)
SLC013	c.1001 + 1G > A; p.(?)	UV5	ACACATG-GC-C (**CEVA**)
SLC018	c.349C > T; p.(Leu117Phe)	UV5	
SLC031	c.1001 + 1G > A; p.(?)	UV5	ACACATG-GC-C (**CEVA**)
SLC032	c.1334 T > G; p.(Leu445Trp)	UV5	
SLC036^a^	c.1246A > C; p.(Thr416Pro)	UV5	ACACATG-GC-C (**CEVA**)
SLC040^a^	c.655_656dup; p.(Phe223Alafs*15)	UV5	GTTCATG-GC-C (**V1-CEVA**)
SLC045	c.1334 T > G; p.(Leu445Trp)	UV5	
SLC048	c.706C > G; p.(Leu236Val)	UV4	
SLC056	c.707 T > C; p(Leu236Pro)	UV5	ACACATG-GC-C (**CEVA**)
SLC071^a^	c.1334 T > G; p.(Leu445Trp)	UV5	GTTCATG-GC-C (**V1-CEVA**)
SLC078	c.304G > C; p.(Gly102Arg)	UV4	ACACATG-GC-C (**CEVA**)
SLC079	c.1001 + 1G > A; p.(?)	UV5	ACACATG-GC-C (**CEVA**)
SLC085	c.706C > G; p.(Leu236Val)	UV4	

Presence of the CEVA haplotype was tested in zeroallelic (M0) and monoallelic (M1) *SLC26A4* cases with a unilateral or bilateral enlarged vestibular aqueduct. *SLC26A4* (NM_000441.1) variants reported in ClinVar as (likely) pathogenic (UV4, UV5) were considered causative, whereas variants reported as (likely) benign or of unknown significance were considered non-causative. In ten individuals, the complete CEVA haplotype was detected (ACACATG-GC-C), whereas in two individuals a shorter version of the haplotype was found, consisting of 9/12 CEVA SNPs (GTTCATG-GC-C; V1). For individuals marked with an ^a^, it could be conclusively determined that the (V1-)CEVA haplotype is present on the *trans SLC26A4* allele

*ACMG* variant classification according to the American College of Medical Genetics and Genomics (ACMG) classification guidelines (Oza et al. 2018), *UV3* uncertain significance, *UV4* likely pathogenic, *UV5* pathogenic

The CEVA haplotype has an AF of 2.8% in the 1000G database (28 in 1006 alleles) (Chattaraj et al. 2017; Genomes Project Consortium et al. 2015), and an AF of 3.3% in an in-house control cohort consisting of 322 healthy unrelated individuals (21 in 644 unphased alleles). This implies a significant enrichment of the CEVA haplotype in our M1 cohort (8 in 32 alleles) compared to the 1000G database (*p* value 5.419*10^–6^) and the control cohort (*p* value 2.187*10^–5^) as determined by a two-sided Fisher’s exact test). The two M1 cases with the V1-CEVA haplotype were not included in this statistical analysis. Also this V1-CEVA allele is significantly enriched in our M1 cohort as only a single V1-CEVA allele is reported in the 1000G database (1 in 1006 alleles) (Chattaraj et al. 2017) (p-value 0.0027). The CEVA haplotype was not found to be significantly enriched in the M0 cohort (2 in 24 alleles). Although the pathogenicity of the CEVA haplotype is unclear, the significant enrichment of the haplotype within this M1 patient cohort and the patient cohorts (M1 and M0) previously described by Chattaraj and co-workers strongly suggests that a pathogenic defect resides within this haplotype (Chattaraj et al. 2017). Because of this strong association of the CEVA haplotype with HL and EVA, we considered the M1 individuals carrying the CEVA or the V1-CEVA haplotype as genetically explained (M1/CEVA), and M0 individuals with the CEVA haplotype (M0/CEVA) as monoallelic in further steps of this study. For six M1 individuals, it could not be conclusively determined whether the CEVA haplotype was present *in trans* with the pathogenic *SLC26A4* variant, as the genetic material of family members was not available (Table 1).

### Whole-genome sequencing reveals potential *SLC26A4* splice and regulatory variants in M1 subjects without the CEVA haplotype

To detect any potentially missed coding or unidentified intronic *SLC26A4* variants or variants located *in cis* regulatory elements of the gene, WGS analysis was performed for all six M1 individuals who could not be genetically explained by the presence of the CEVA haplotype. Additionally, WGS analysis was performed for the two M0/CEVA individuals. In none of these eight cases, SVs overlapping with the *SLC26A4* gene were identified by WGS.

To identify any variants with a potential effect on splicing, the deep-learning algorithm SpliceAI was employed (Jaganathan et al. 2019). In two M1 individuals (SLC048 and SLC085), a rare heterozygous potentially splice altering *SLC26A4* variant was identified (Table 2). For both variants, the predicted splice defect was investigated using an in vitro splice assay performed in HEK293T cells. For SLC048, a canonical splice site variant (c.1342-2A > C), that was overlooked during prescreening efforts, was predicted to remove the splice acceptor site. This variant was previously reported in a study performed by Van Beeck Calkoen and coworkers and in ClinVar (van Beeck Calkoen et al. 2019). Indeed, the splice assay revealed loss of the acceptor site and usage of an alternative splice acceptor site located thirteen nucleotides downstream (Online Resource Figure S1A). This leads to the formation of an out-of-frame exon 12 and premature protein truncation [p.(Ser448Leufs*3)]. Based on these results, the variant was classified as pathogenic according to the ACMG guidelines (Oza et al. 2018). In SLC085, a synonymous variant (c.471C > T, classified as likely benign in ClinVar) was identified in exon 5. SpliceAI predicts that this variant strengthens an alternative splice acceptor site (27 nucleotides downstream of the variant). Indeed, an in vitro splice assay confirmed that the alternative splice acceptor site is used, which leads to the partial deletion of exon 5 and a truncated protein [p.(Gly139Alafs*6)] (Online Resource Figure S1B)**.** Therefore, this variant is now classified as pathogenic according to the ACMG classification guidelines (Oza et al. 2018). The observed splice defect resulting from this synonymous variant underlines the importance of evaluating potential splice effects of all rare variants in coding sequences, using in silico prediction splice tools. We considered the two identified splice variants as pathogenic and the HL of the two individuals as genetically explained, thus M2.

**Table 2:** WGS revealed two heterozygous splice variants in *SLC26A4*

Case	Class	Genome	cDNA	Protein	In-house AF (%)	gnomAD AF (%)	CADD_PHRED	SIFT	PPH2	MutationTaster	SpliceAI	ACMG
SLC048	M1	Chr7:107335064A > C	c.1342-2A > C	p.Ser448Leufs*3	0.00	–	**21.7**	NA	NA	NA	**0.99 (AS loss)**	UV5
SLC085	M1	Chr7:107314664C > T	c.471C > T	p.Gly139Alafs*6, =	–	0.00	0.725	NA	NA	NA	**0.59 (AS gain)**	UV5

Whole genome sequencing (WGS) revealed two potentially splice altering variants in *SLC26A4*. Variants are selected based on an allele frequency of ≤ 0.5% in gnomAD and the in-house database. Scores that meet the thresholds for pathogenicity as described in the methods section are indicated in bold. The predicted effect on splicing was confirmed in an in vitro splice assay that was performed in HEK293T cells (Fig. S1)

*Genome* genomic position according to GRCh37/hg19, *In-house AF* allele frequency (%) in an in-house database (~ 7500 exomes), *GnomAD AF* allele frequency (%) in gnomAD database V.2.1.1, *CADD_PHRED* Combined Annotation Dependent Depletion PHRED score, *SIFT* scale-invariant feature transform, *PPH2* PolyPhen-2 score, *MutationTaster (prob)* MUTATIONTASTER score with probability (0–1), *spliceAI* splicing prediction score, *AS* acceptor site, *ACMG* variant classification according to the American College of Medical Genetics and Genomics (ACMG) classification guidelines (Oza et al. 2018), *UV5* pathogenic, *NA* not applicable

To explore variants that are potentially located within a *cis*-regulatory element of *SLC26A4*, we extracted all (predicted) human enhancer and promoter elements that are associated with the *SLC26A4* gene from the GeneHancer (Fishilevich et al. 2017) and EnhancerAtlas (Gao and Qian 2020) databases (Online Resource Table S5). GeneHancer V5 is a collection of both predicted and experimentally validated enhancer-to-gene and promoter-to-gene interactions, based on information integrated from multiple resources: ENCODE (Dunham et al. 2012), Ensembl (Zerbino et al. 2015), FANTOM5 (Andersson et al. 2014), VISTA (Visel et al. 2007), dbSuper (Khan and Zhang 2016), EPDnew (Dreos et al. 2013), UCNEbase (Dimitrieva and Bucher 2013) and CraniofacialAtlas (Wilderman et al. 2018). For each regulatory element, a gene interaction score (> 7) and element confidence score (> 0.7) are provided. The EnhancerAtlas V2 is a database providing enhancer annotations in different species based on experimental datasets determined in several tissues and cell types.

WGS data were analyzed for variants located within these elements and two rare potentially regulatory variants (Chr7:107220628C > A, Chr7:107384987C > G) were identified in two M1 individuals (SLC002 and SLC045) (Online Resource Table S6). Both variants are located in a predicted enhancer element of *SLC26A4* according to GeneHancer. We did not find any strong indication of a functional effect for the two variants based on (nucleotide) conservation scores [PhyloP, UCSC genome browser (Kent et al. 2002)) or loss of transcription factor binding sites [JASPAR database (Fornes et al. 2020)]. Therefore, the variants were considered non-pathogenic, although only a reporter assay can completely exclude a potential regulatory effect of the variants on *SLC26A4* expression.

### A *FOXI1* missense variant is revealed in three unrelated index cases

Several studies have suggested a potential digenic inheritance for *SLC26A4* variants and variants in *KCNJ10* and *FOXI1* (Pique et al. 2014; Yang et al. 2009, 2007). Additionally, a more recent study suggested digenic inheritance with pathogenic variants in *EPHA2* (Li et al. 2020). We screened all remaining genetically unexplained individuals (M1, M0/CEVA and M0) for variants in these genes with an AF ≤ 5% (gnomAD V2.1.1). In cases for which only MIP sequencing data was available, coding regions and exon–intron boundaries of *FOXI1* and the regions harboring the reported pathogenic variants in *EPHA2* (c.1063G > A; p.(G355R), c.1532C > T; (p.T511M), NM004431.4) were analyzed using Sanger sequencing. In three individuals (SLC039; M0/CEVA, SLC052; M0 and SLC069; M0) a c.677C > T (p.(Thr226Ile)) *FOXI1* (NM_012188.4) missense variant was identified (Table 3). The variant was not identified in any of the M1/CEVA or the two M2 cases. *FOXI1* encodes the Forkhead transcription factor FOXI1, a key transcriptional regulator of *SLC26A4* (Yang et al. 2007). Segregation analysis has confirmed that the *FOXI1* variant is not co-inherited with the CEVA allele in individual SLC039, which is in line with digenic inheritance*.* The Thr226 residue is located outside of the conserved forkhead DNA-binding domain of FOXI1 (amino acids 94-211) (Yang et al. 2007) and none of the in silico tools used for analysis predicted a deleterious effect of the c.677C > T variant. Nevertheless, the variant is enriched in individuals diagnosed with HL and EVA (3 in 56 alleles in the study cohort versus 165 in 26.590 alleles of the in-house WES cohort, *p* value 0.0004), and we consider the c.677C > T *FOXI1* variant as an interesting candidate for functional validation.

**Table 3:** Rare variants identified in *EPHA2*, *FOXI1* and *KCNJ10*

Case	Class	Gene	Transcript	cDNA	Protein	In-house AF (%)	gnomAD AF (%)	CADD_PHRED	SIFT	PPH2	Mutation Taster	SpliceAI	ACMG
SLC017	M0	*EPHA2*	NM_004431.4	c.2627G > A	p.(Arg876His)	2.36	1.70	**32**	**0**	**0.769**	NA	0.03	UV2
SLC039	M0/CEVA	*FOXI1*	NM_012188.4	c.677C > T	p.(Thr226Ile)	0.56	0.37	11	0.14	0.109	P	0.03	UV2
SLC052	M0	*EPHA2*	NM_004431.4	c.1941G > T	p.(Thr647 =)	1.09	0.55	7.309	NA	NA	NA	0.05	UV2
SLC052	M0	*EPHA2*	NM_004431.4	c.1896G > A	p.(Leu632 =)	0.76	0.05	3.197	NA	NA	NA	0.05	UV2
SLC052	M0	*FOXI1*	NM_012188.4	c.677C > T	p.(Thr226Ile)	0.56	0.37	11	0.14	0.109	P	0.03	UV2
SLC069	M0	*FOXI1*	NM_012188.4	c.677C > T	p.(Thr226Ile)	0.56	0.37	11	0.14	0.109	P	0.03	UV2

Available sequencing datasets of monoallelic (M1, M0/CEVA) and zeroallelic (M0) individuals were screened for variants in *EPHA2*, *FOXI1* and *KCNJ10* with an allele frequency of ≤ 5% in gnomAD (V.2.1.1). Scores that meet the thresholds for pathogenicity as described in the methods section are indicated in bold

*In-house AF* allele frequency (%) in in-house database (~ 7500 exomes), *GnomAD AF* allele frequency (%) in gnomAD database V.2.1.1, *CADD_PHRED* Combined Annotation Dependent Depletion PHRED score, *SIFT* Scale-Invariant Feature Transform, *PPH2* PolyPhen-2 score, *MutationTaster (prob)* MutationTaster score with probability (0–1), spliceAI splicing prediction score, *ACMG* variant classification according to the American College of Medical Genetics and Genomics (ACMG) classification guidelines (Oza et al. 2018), *UV2* likely benign, *NA* not available, *P* polymorphism

In case SLC017, a heterozygous missense variant in *EPHA2* was detected (c.2627G > A) [p.(Arg876His)]. Although the variant is predicted to be pathogenic by in silico prediction tools, it has a relatively high AF of 1.70% (gnomAD) and 2.36% (in-house database) and is classified as likely benign according to the ACMG classification guidelines. Because the variant was only found in an M0 *SLC26A4* case, a potential digenic inheritance of pathogenic *SLC26A4* variants and the newly identified *EPHA2* variant could not be addressed.

To summarize, the CEVA haplotype or a short CEVA haplotype (V1-CEVA) was detected in 12 of the 28 index cases (16 M1, 12 M0) that were included in our study (Fig. 1). In two individuals (M1), an *SLC26A4* splice variant was identified using WGS. After performing these genetic analyses by which the enrichment of the (V1−)CEVA haplotype in M1 cases was demonstrated, we consider the HL in 12 individuals to be associated with *SLC26A4* defects and these subjects to be genetically explained (2 M2, 10 M1/CEVA), six individuals are considered M1 (4 M1, 2 M0/CEVA), and ten individuals are still considered M0. Additionally, in three individuals (1 M0/CEVA, 2 M0) a potentially pathogenic variant in *FOXI1* was found.

### Determination of boundaries of the CEVA haplotype

**Fig. 2:**
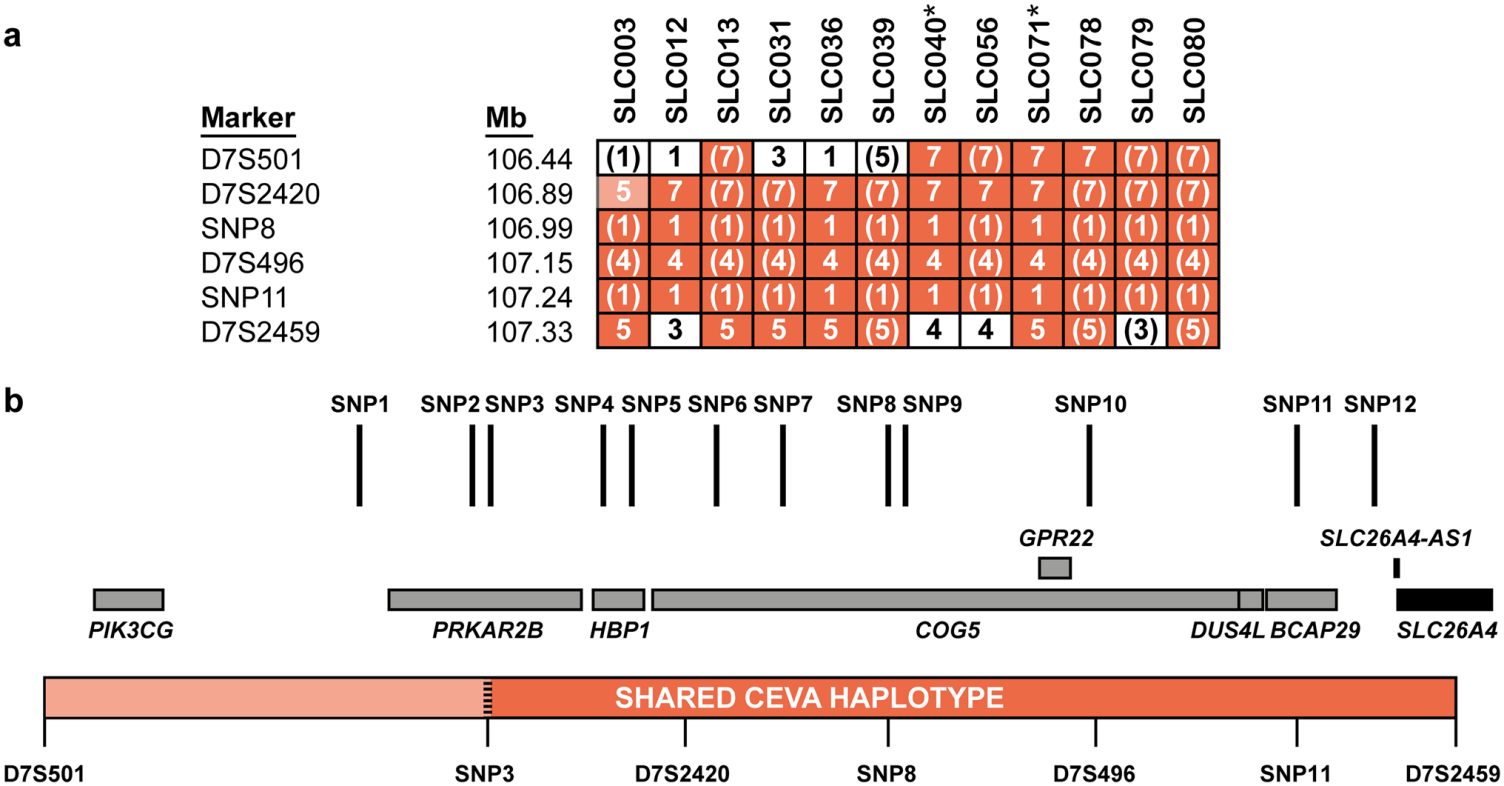
Determination of the boundaries of the shared CEVA haplotype. **a** The CEVA haplotype was detected in 10 individuals, in an additional 2 individuals (SLC040 and SLC071, indicated with *), a smaller haplotype was found, termed V1-CEVA. To determine the boundaries of the CEVA haplotype, VNTR marker analysis was performed. The shared haplotype (0.89 Mb, CEVA; 0.57 Mb V1-CEVA is marked in orange. For marker D7S2420 (light-orange) a deviating CA-repeat length was determined in SLC003. Nevertheless, the marker is still considered to be potentially part of the shared haplotype as a change or repeat length cannot be excluded. Genomic positions (Mb) are according to the UCSC Genome Browser (GRCh37/hg19). **b** A schematic overview of the identified shared CEVA haplotype (D7S501-D7S2459). Positions of the CEVA-associated SNPs and the genes located within the haplotype region (CEVA, D7S501-D7S2459; V1-CEVA, SNP3-D7S2459) have been indicated. All SNPs are located within intronic or intergenic regions. Genomic positions of the CEVA-associated SNPs are provided in Table S7. *SLC26A4* (NM_000441.1) is only partially included (exons 10/21) in the shared haplotype

To identify the true pathogenic defect located on the CEVA haplotype, an in-depth analysis of this genomic region was performed. Firstly, the exact boundaries of the genomic region shared by CEVA haplotype carriers were determined using VNTR marker analysis. For two individuals with the complete CEVA haplotype and the two subjects with the V1-CEVA haplotype, DNA samples of family members were available, allowing reliable determination of the marker alleles located within the haplotype. A shared haplotype of 0.89 Mb delimited by markers D7S501 and D7S2459 was identified (Fig. 2 and Online Resource Figure S2). Although the V1-CEVA haplotype shares the marker alleles with the complete CEVA haplotype, the absence of SNPs 1–3 potentially delimits the shared haplotype even more (0.57 Mb, CEVA SNP 3-D7S2459). The remaining eight individuals with the complete CEVA haplotype share identical marker alleles in the 0.89 Mb-sized region, although they could not be conclusively assigned to the haplotype as no segregation analysis could be performed. For individual SLC003, a deviating repeat length was identified for marker D7S2420. As we cannot exclude a rare event to be responsible for the change in allele length, this marker was still considered part of the shared CEVA haplotype.

### Short-read WGS did not reveal a pathogenic defect on the CEVA haplotype

Because of the significant enrichment of the CEVA haplotype in M1 cases, we hypothesized that the subjects with the CEVA haplotype share a yet elusive pathogenic defect. To identify this defect on the CEVA haplotype, short-read WGS was performed in two individuals (SLC012 and SLC036) carrying the CEVA haplotype *in trans* with a pathogenic variant in *SLC26A4* (M1/CEVA). All heterozygous variants with an AF ≤ 5% in gnomAD that were shared between the two individuals and located within the determined boundaries of the CEVA haplotype were analyzed (Online Resource Table S7). In total, 20 shared variants remained and included the 12 original SNPs that previously defined the CEVA haplotype (Chattaraj et al. 2017). Sixteen of the shared variants are located in intronic regions, but for none of them, a significant effect (score ≥ 0.1) on splicing is predicted by SpliceAI. Two variants are located within a *cis-*regulatory element of *SLC26A4* according to the GeneHancer database, however, these also show overlap with a long interspersed nuclear element (LINE) repeat element. One variant (CEVA SNP9) has a high nucleotide evolutionary conservation score (PhyloP, 2.769 [range − 14, 3]). No SVs or CNVs were detected within or overlapping with the CEVA haplotype and shared by the two individuals.

Regions harboring heterozygous variants with an AF ≤ 5% in gnomAD that were not shared between SLC012 and SLC036 had sufficient coverage to exclude that these variants were only called in one of the subjects but present in both of them. None of the variants identified in either SLC012 or SLC036 were within the *SLC26A4* gene or were obviously deleterious. SVs and CNVs within the CEVA boundaries were analyzed separately for the two subjects which did not reveal any of such variants that were not shared by the two studied subjects. To fully exclude that the CEVA haplotype harbors different pathogenic variants in the studied individuals, a study design including short- and long-read WGS in several nuclear families has to be applied.

### Optical genome mapping & long-read sequencing

To investigate the possibility that SVs were missed using short-read sequencing, optical genome mapping (Bionano Genomics) was performed using ultra-high molecular weight DNA isolated from peripheral blood cells of individual SLC012 (M1/CEVA). Optical genome mapping identified a total of 6,565 SVs, of which none were within the CEVA region (D7S501-D7S2459; chr7:106,440,266–107,360,254). Two SV calls (both calling the same 2196 bp insertion between chr7:107,367,549 and 107,373,585) were located just outside this region (Online Resource Figure S3A). This insertion was also called in 100% of our current optical genome mapping control cohort (Levy-Sakin et al. 2019), strongly suggesting that this reflects a reference problem rather than a real SV. Additionally, there were 22 CNV calls, of which none were within the CEVA region.

Subsequently, PacBio long-read sequencing was performed on genomic DNA isolated from individual SLC079 (M1/CEVA; *in trans* status unknown). SV analysis of the long-read sequencing data revealed a total of 55,205 SVs, of which 12 within the CEVA region. After careful interrogation of the SVs, all of them were considered false positives based on SV length, and the presence of the SVs in an in-house control dataset. The CEVA haplotype region was also manually inspected in the IGV software, which did not reveal any indications for potential SVs (Online Resource Figure S3B). Interestingly, the insertion event that was detected with optical genome mapping and located just outside the CEVA region was also present in the long-read sequencing data (chr7:107,370,573, 1612 bp insertion). This insertion was also present in available in-house control sequencing data, supporting the hypothesis the variant concerns a reference problem and is not a true SV.

### A comparable severity of hearing loss in the M1/CEVA and M2 cohorts

**Fig. 3:**
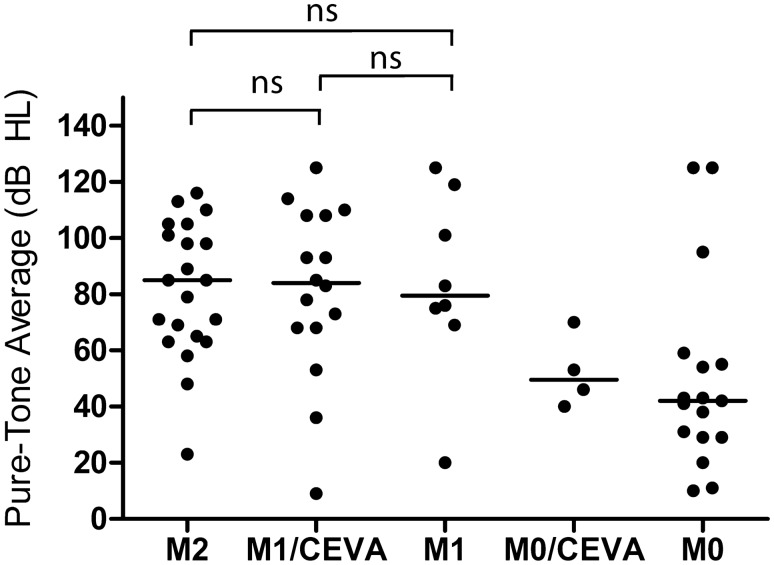
Results of audiometric evaluation in affected individuals. PTA_0.5–4 kHz_ for ears with an EVA. Each dot represents the hearing level of an ear with an enlarged vestibular aqueduct, allocated to genotype class (M2, M1/CEVA, M1, M0/CEVA and M0). The M1/CEVA group also includes subjects with an M1/V1-CEVA genotype. For an objective comparison, the same methods as used by Chao et al. (2019) were applied

As the CEVA haplotype was reported to be associated with a less severe HL phenotype than variants in the protein-coding or splice site regions of *SLC26A4* (Chao et al. 2019), we addressed genotype–phenotype correlations in our cohort. We were able to retrieve pure tone audiometry of all subjects except for SLC071; for this subject, complete audiometry from only one ear was available (Online Resource Figure S4). The original CT or MRI scans of subjects SLC018 and SLC032 could not be retrieved. However, written reports of the imaging were available. Data on thyroid gland function were not consistently available and were therefore not included in this study. We applied the methods of Chao et al. (2019) to compare the severity of HL between four subject groups (M0, M1, M1/CEVA, and M2 Fig. 3, Table 4) (Chao et al. 2019). The M1/CEVA group includes the M1/V1-CEVA subjects. Bilateral EVA was present in 7 of 10 (70%) M1/CEVA subjects, in all 4 M1 subjects without the CEVA haplotype, and 7 of 10 (70%) M0 subjects without the CEVA haplotype. All 11 M2 subjects (reference cohort, SLC048 and SLC085) had bilateral EVA. The median pure tone audiometry in the M2 group (85 dB HL, *n* = 20) was not significantly different from that of the M1/CEVA group (84 dB HL, *n* = 16) and the M1 group (79.5 dB HL, *n* = 8) (*p* values 0.8300 and 0.7142, respectively, all adjusted for age). Also, no difference was observed between the M1/CEVA group and the M1 group (*p* = 0.8782). In contrast, when we compare the M2 and M1/CEVA groups with the M0 group, we observed significant differences in the severity of HL (*p* = 0.0015 and *p* = 0.0135, respectively). When compared to Chao et al., subjects in our study displayed a similar degree of median HL in the M2 group (86.3 and 85 dB in Chao et al. 2019 and the present study, respectively), more severe HL in the M1/CEVA group (47.5 and 84 dB, respectively) and less severe HL in the M0 group (54.4 and 42 dB HL, respectively). Slight age differences were seen between the groups presented in Chao et al. 2019 and those in the current study (Online Resource Table S8). Chao and co-workers did not report audiometric data for the M1 group without the CEVA haplotype *in trans*, presumably due to the small sample size. Overall, in contrast to the study by Chao et al. (2019), the present study showed that subjects with biallelic pathogenic variants in the coding regions and splice sites of *SLC26A4* have a degree of HL that is similar to that of subjects with a monoallelic *SLC26A4* variant and the CEVA haplotype. Due to the small sample size, we could not test the hypothesis that the CEVA haplotype acts as a modifier in M0 subjects as reported previously (Chao et al. 2019).

**Table 4:** Clinical evaluation of affected individuals

Class	Case	Gender	Age of onset (year)	Otoscopic examination	Newborn hearing screening	Motor development		Imaging		Audiometric evaluation		
										Subject age (yr)	PTA (0.5-4 kHz)	
								CT	MRI		R	L
M0	SLC014	M	PC	N	3rd time pass	Delayed		bil EVA		4	> 120	55
	SLC015	F	PS	Myringosclerosis	N	NR			uni EVA R	25	29	66
	SLC017	M	PC	N	NA	NR		bil EVA		5	10	59
	SLC043	F	PC	N	N	NR		uni EVA L		17	10	95
	SLC052	F	33	N	NA	NR		bil EVA		33	20	> 120
	SLC069	F	15	N	NA	NR		bil EVA		19	43	42
	SLC070	M	PC	N	NA	N			bil EVA	19	43	31
	SLC073	M	U	L: atelectatic middle ear, retracted eardrum	NA	NR			uni EVA R	12	11	18
	SLC084	F	PC	N	3rd time pass	Delayed		bil EVA		6	29	38
	SLC086	M	PC	N	N	Delayed		bil EVA		6	54	41
M0/CEVA	SLC039	F	2–4	N	NA	NR			bil EVA	24	46	70
	SLC080	F	5	N	N	N		bil EVA		6	40	53
M1	SLC002	F	U	N	NA	NR		bil EVA		18	83	76
	SLC018	M	PC	N	NA	N		bil EVA^a^		16	75	101
	SLC032	F	PC	N	NA	NR		bil EVA^a^		59	119	> 120
	SLC045	F	PC	N	N	N		bil EVA		7	20	69
M1/CEVA	SLC003	M	15	N	NA	NR	uni EVA R			17	83	− 1
	SLC012	M	PC	N	NA	NR	bil EVA			17	> 120	53
	SLC013	F	U	N	NA	NR	bil EVA			22	36	9
	SLC031	M	PS	N	2nd time pass	N	uni EVA L			16	0	68
	SLC036	F	PC	N	NA	NR	bil EVA			20	73	68
	SLC040	M	U	N	U	N			uni EVA L	7	5	78
	SLC056	M	PC	N	NA	NR	bil EVA			14	108	93
	SLC071	M	PC	N	N	N	bil EVA			3	85	NT
	SLC078	F	PC	N	NA	U	bil EVA			10	114	93
	SLC079	F	C	N	R	N	bil EVA			2	110	108
M2	SLC048	M	PC	N	NA	NR	bil EVA			8	105	71
	SLC085	M	C	N	R	N	bil EVA			2	23	85
	SLC087	F	C	N	R	Delayed			bil EVA	5	65	63
	SLC088	F	4	N	NA	N			bil EVA	17	85	105
	SLC089	M	U	N	U	U			bil EVA	10	58	98
	SLC090	F	3	N	NA	U			bil EVA	41	116	110
	SLC091	F	2–4	N	N	Delayed	bil EVA			12	63	69
	SLC092	F	C	N	R	N	bil EVA			4	79	48
	SLC093	M	PC	N	U	Delayed			bil EVA	8	71	98
	SLC094	M	PC	Sclerotic eardrum L	NA	U	bil EVA			37	101	113
	SLC095	F	C	N	R	N	bil EVA			1	NT	89

Age of onset (AoO), age of onset in years as reported by the subjects. Subject age, the age at which the audiometric data of the last two columns were obtained, in general the last audiogram. Newborn hearing screening was introduced in the Netherlands in 2006

*Y* years, *PTA* pure tone average mean of 0.5 1 2 and 4 kHz air conduction thresholds, *M* male, *F* female, *R* right, *L* left, *PC* age of onset of HL is presumably congenital based on anamnesis, *C* age of onset of HL is congenital based on newborn hearing screening, *PS* subject reported onset of HL during primary school exact age unknown, *NR* not reported, *NT* not tested, *N* no abnormalities, *R* refer in newborn hearing screening failed in test, *U* unknown, *CT* computed tomography, *MRI* magnetic resonance imaging, *uni EVA L/R* unilateral enlarged vestibular aqueduct in left or right ear, *bil EVA* bilateral enlarged vestibular aqueduct

^a^Only written report available

## Discussion

In this study, we investigated 28 genetically unexplained Dutch index cases with HL and a unilateral or bilateral EVA. To elucidate the missing heritability in monoallelic *SLC26A4* cases, who represent 14–31% of subjects with HL and EVA (Azaiez et al. 2007; Mey et al. 2019), extensive genomic analyses as well as phenotyping were performed. Important findings in this study were (1) the enrichment of a shared (V1−)CEVA haplotype in M1 *SLC26A4* cases, (2) two *SLC26A4* splice variants and (3) the identification of a *FOXI1* variant in three subjects suggesting a contribution of this variant to the etiology of HL and EVA. Furthermore, the genotype–phenotype analyses revealed that the severity of the HL associated with biallelic variants (M2) in *SLC26A4* is comparable to the HL associated with a monoallelic variant in *SLC26A4* with or without the CEVA haplotype (M1 and M1/CEVA).

For six M1 individuals, it could not be conclusively determined whether the CEVA haplotype was present *in trans* with the (likely) pathogenic *SLC26A4* variant, as no genetic material of family members could be obtained. However, we anticipated that most if not all of the six M1 cases carry the CEVA haplotype *in trans* with the *SLC26A4* variants because it seems highly unlikely that the *SLC26A4* variants all have occurred on an allele with a frequency of < 3% in the population (Chattaraj et al. 2017). Furthermore, the co-occurrence of two partial CEVA haplotypes that together exactly mimic a heterozygous CEVA haplotype in 6 of 16 individuals is highly unlikely as the frequencies of partial CEVA haplotypes in the European population are all (far) below 1% (Chattaraj et al. 2017). The same holds true for the two M0/CEVA cases for whom we could not determine the phase of the 12 SNPs in the CEVA haplotype.

In two cases, the V1-CEVA haplotype was identified. This smaller CEVA haplotype was also reported previously in a single M1 case by Chattaraj and coworkers and likely refines the CEVA haplotype. Alternatively, the V1-CEVA haplotype harbors a different genetic defect. The shared VNTR marker alleles of the V1-CEVA and the CEVA haplotype suggest that V1-CEVA refines the boundaries of the shared genomic region to 0.57 Mb.

We anticipated that a pathogenic variant co-segregates with the CEVA haplotype. Therefore, we subjected the shared genomic region to extensive genomic analyses that included WES, short- and long-read WGS, and optical genome mapping, to reveal any potential variants missed or misinterpreted in earlier studies. None of the applied sequencing or imaging techniques revealed rare SVs that overlap or are present within the CEVA haplotype. In the light of the proven accuracy and efficacy of especially optical genome mapping and long-read sequencing in SV detection (Chaisson et al. 2019), we deem it unlikely that any SVs within the CEVA region escaped detection. Additionally, we evaluated all SNVs with an AF ≤ 5% (gnomAD) present within the region for predicted regulatory or splice altering effects but for none of the 20 SNVs a potential effect was predicted by SpliceAI. Two SNVs overlap with a potential regulatory element of *SLC26A4* (GeneHancer, EnhancerAtlas), and one variant is present within the intronic regions of this gene. However, all three variants are located within a highly repetitive element (LINE). Although little is known about the effects of genetic variation within LINE elements, a potential effect on the methylation landscape and consequently gene expression levels has been suggested (Xie et al. 2009) and such an effect can therefore not be excluded for the three indicated variants. For the remaining SNVs, no potential effects on transcript splicing or gene regulation were predicted. Nevertheless, we cannot rule out combinatory effects of the SNVs, since they are all located in *cis*. A thorough experimental (multi-omic) analysis is required to optimally assess the effects of the identified variants. RNA studies can be performed to detect quantitative or qualitative changes affecting the *SLC26A4* transcripts. A defect observed on the RNA level could provide valuable insights that may point towards the true pathogenic defect, and prioritize one, or a combination, of the variants on the CEVA allele. However, *SLC26A4* is not or at extremely low levels expressed in readily accessible patient cell types (e.g., fibroblasts and blood cells). The same holds true for induced pluripotent stem cells or otic progenitor cells (Hosoya et al. 2017). However, Hosoya and co-workers have successfully developed a protocol that allows the differentiation of otic progenitor cells into outer sulcus-like cells that express *SLC26A4* at high levels. This protocol could potentially be a powerful tool to evaluate the consequences of CEVA haplotype at the RNA level.

*SLC26A4* is not the only gene present within the CEVA haplotype, which also spans *BCAP29*, *COG5*, *DUS4L*, *HBP1*, *PIK3CG,* and *PRKAR2B*. For none of these genes, pathogenic variants associated with (syndromic) HL have been reported, nor has a function in the inner ear been described. The majority of the CEVA-associated SNVs (16/20) are located within an intronic region of these genes, however, for none of these variants a splice altering effect is predicted by SpliceAI.

Since the genetic defect on the CEVA haplotype could not be pinpointed by the genetic analyses, we could not determine whether the AF of the defect is lower than that of the CEVA haplotype and more in line with the expected frequency based on the prevalence of HL (1:1,000 newborns (Morton and Nance 2006)) and the genetic heterogeneity of the condition. Alternatively, the CEVA haplotype could be considered a hypomorphic allele, of which the penetrance depends on the contribution of other co-existing (common) variants.

Not all M0 or M1 *SLC26A4* cases could be genetically explained by the presence of the CEVA haplotype. Therefore, digenic inheritance with variants in *EPHA2, FOXI1,* and *KCN10* was also explored as a potential explanation for the missing heritability. Digenic inheritance of defects in *SLC26A4* and *EPHA2* has recently been reported in two Japanese Pendred syndrome cases (Li et al. 2020). A c.1063G > A [p.(Gly355Arg)] and a c.1532C > T [p.(Thr511Met)] variant in *EPHA2* were each found ‘*in trans’* with a reported pathogenic variant in *SLC26A4* (Deafness Variation Database (Azaiez et al. 2018)). EPHA2 was identified as a binding partner of pendrin, with a crucial role in regulating pendrin localization (Li et al. 2020). The identified variants in *EPHA2* were predicted to be pathogenic by several in silico predictions tools. However, the c.1532C > T variant has a relatively high allele frequency of 3.03% in the East Asian population, including 11 homozygotes (gnomAD). Yet, in the present study, we did not obtain indications for the digenic inheritance of variants in *SLC26A4* and *EPHA2* in subjects with HL and EVA. Besides for *EPHA2*, a digenic mechanism has also been reported and debated for variants in *SLC26A4* and *KCNJ10* or *FOXI1*, with currently no consensus (Jonard et al. 2010; Landa et al. 2013; Pique et al. 2014; Yang et al. 2009, 2007). FOXI1 is a transcriptional regulator of *SLC26A4* (Yang et al. 2007). We identified a c.677C > T (p.(Thr226Ile)) *FOXI1* variant in three subjects (2 M0/*FOXI1* and 1 M0/CEVA/*FOXI1*). This variant was previously detected in an individual diagnosed with Pendred syndrome and a monoallelic pathogenic *SLC26A4* variant (Pique et al. 2014). The variant has an allele frequency of 0.71% in non-Finnish Europeans (gnomAD) and affects an amino acid residue located outside the DNA-binding domain but close to the nuclear localization signal (NucPred (Brameier et al. 2007)). Previously reported pathogenic *FOXI1* variants have been shown to affect the DNA-binding properties of the protein (Enerbäck et al. 2018). We speculate that a variant affecting the localization motif of the protein could potentially have a loss of function effect as well. Although the variant is classified as likely benign according to the ACMG classification guidelines, we identified the variant three times in our cohort of genetically unexplained *SLC26A4* cases and combined with the fact that it has been reported in a previous study (Pique et al. 2014), this suggests that the variant might actually contribute to the etiology of HL and EVA although not in a monogenic pattern. Interestingly, in *Foxi1*^*−/−*^ mice, the expansion of the endolymphatic compartment and an audio-vestibular phenotype was observed (Hulander et al. 2003). In situ hybridization of the endolymphatic duct and sac of these mice revealed complete absence of *Slc26a4* mRNA expression. Functional studies, among which cellular localizations assays, are warranted to evaluate the effect of the c.677C > T *FOXI1* variant. We did not identify likely pathogenic variants in *KCNJ10* (AF ≤ 5%) in our cohort.

WGS did not reveal strong candidate regulatory variants based on data derived from enhancer databases and transcription factor binding site predictions. Nevertheless, interpretation of regulatory variants is still considered complex and is limited by the lack of available epigenetic datasets for the inner ear. In addition, no SVs overlapping with *SLC26A4* were detected using WGS, suggesting a limited contribution of SVs to the mutational landscape of *SLC26A4*. This is in line with earlier observations described in literature (Liu et al. 2021; Pique et al. 2014). For the monoallelic cases (M1, M0/CEVA), no long-read sequencing or optical genome mapping was performed. As it is generally accepted that most SVs could not be accurately detected using short-read sequencing approaches only (Chaisson et al. 2019), it cannot be excluded that causative SVs are present but missed due to technical limitations.

The present study did not confirm that the CEVA allele is associated with a milder HL compared to *SLC26A4* variants affecting the protein-coding sequences, as indicated by Chao et al. (Chao et al. 2019). They discerned a significantly milder HL in their cohort of M1/CEVA subjects (*n* = 20 ears, median 47.5 dB HL) than we have seen in our cohort of M1/CEVA subjects (*n* = 16 ears, median 84 dB HL). A possible explanation for this discrepancy could be the progression of HL combined with a ~ 5-year difference in average age between the cohorts (7.5 and 12.8 years, respectively). Progression of HL is seen in up to 39.6% of EVA-ears (Alemi and Chan 2015), with progression rates of ~ 3.5 to  ~ 5.5 dB/year (Govaerts et al. 1999; Jackler and de la Cruz 1989). On the other hand, the older subjects in our M1/CEVA cohort show less severe HL than the younger subjects, which is questioning the relationship with age. Furthermore, there is also an average age difference of 5 years between the M2 groups in both studies (13.2 years and 18.4 years, respectively), while the severity of HL is comparable (85 and 86.3 dB HL, respectively).

The reported variability of the auditory phenotype associated with EVAs (Arjmand and Webber 2004; Gopen et al. 2011; Griffith and Wangemann 2011) may be another explanation for the observed differences in severity of HL in both studies. In literature, many prognostic factors such as genotype, EVA size and morphology, age, head trauma, and gender are reported as underlying explanations for this variability, although some of these studies draw contradicting conclusions (Alemi and Chan 2015; Archibald et al. 2019; Ascha et al. 2017; Gopen et al. 2011; Miyagawa et al. 2014; Rah et al. 2015; Saeed et al. 2021). In the same line, Song et al. reported intrafamilial differences in the severity of hearing loss in siblings with the same biallelic variants in *SLC26A4* (Song et al. 2014). Larger sample sizes are needed to confirm or reject the hypothesis that the CEVA haplotype is associated with a milder HL phenotype.

The significant difference in HL severity between the M2 and M1/CEVA groups versus the M0 group suggests that *SLC26A4* defects have a prognostic value which can be strengthened in the future by the identification of the underlying genetic defects in subjects of the M0 group.

In conclusion, the HL and EVA in 12 of the 28 studied subjects could be associated with *SLC26A4*. In addition, we have identified genetic factors that might (partially) explain the phenotype in four additional subjects. However, we could not pinpoint the genetic defect that is present in the CEVA haplotype. The arrival of third-generation sequencing techniques, the expansion of epigenetic and transcriptomic datasets and the increasing understanding of non-coding, structural, and regulatory variants will aid in solving the missing heritability in *SLC26A4* in the coming years. This is of great importance for counseling patients about the underlying cause and expected prognosis of their HL. Furthermore, as variants in *SLC26A4* are a frequent cause of HL (Sloan-Heggen et al. 2016), it is an interesting target for the development of a genetic therapy (Kim et al. 2019). Although the involved molecular defect of the CEVA haplotype is still not resolved, the high prevalence of the CEVA haplotype suggests that a significant portion of monoallelic *SLC26A4* cases can be associated with *SLC26A4* defects by testing for the presence of this haplotype.

## Supplementary Information

Below is the link to the electronic supplementary material.

Supplementary file1 (PDF 3168 KB)

## Data Availability

Not applicable.
