# Supplementary material for: Exploring the missing heritability in subjects with hearing loss, enlarged vestibular aqueducts, and a single or no pathogenic SLC26A4 variant
Source: Hum Genet. 2021 Aug 19;141(3-4):465–84. doi: 10.1007/s00439-021-02336-6 (PMC9035008; doi:10.1007/s00439-021-02336-6)
Supplement: Supplementary file 1 — Supplementary file1 (PDF 3168 KB) [file 439_2021_2336_MOESM1_ESM.pdf]

SUPPLEMENTARY TABLES

Table S1: Genotype of reference cohort with biallelic pathogenic variants in *SLC26A4*

	Allele 1		Allele 2	
**Case**	**Variant**	**ACMG**	**Variant**	**ACMG**
SLC087	c.1147del; p.(Gln383Argfs*49)	UV5	c.1147del; p.(Gln383Argfs*49)	UV5
SLC088	c.2T>C; p.(Met1?)	UV5	c.707T>C; p.(Leu236Pro)	UV5
SLC089	c.890del; p.(Pro297Glnfs*6)	UV5	c.1246A>C; p.(Thr416Pro)	UV5
SLC090	c.1001+1G>A; p.(?)	UV5	c.1001+1G>A; p.(?)	UV5
SLC091	c.1225C>T; p.(Arg409Cys)	UV5	c.707T>C; p.(Leu236Pro)	UV5
SLC092	c.1694G>A; p.(Cys565Tyr)	UV5	c.707T>C; p.(Leu236Pro)	UV5
SLC093	c.754T>C; p.(Ser252Pro)	UV4	c.1174A>T; p.(Asn392Tyr)	UV5
SLC094	c.2048T>C; p.(Phe683Ser)	UV5	c.707T>C; p.(Leu236Pro)	UV5
SLC095	c.1246A>C; p.(Thr416Pro)	UV5	c.707T>C; p.(Leu236Pro)	UV5

Genotype of a control cohort of nine subjects with two (likely) pathogenic variants in the coding or splice site regions of *SLC26A4* and a Pendred syndrome phenotype. Segregation analysis to confirm biallelic occurrence of the variants could be carried out in all subjects, except for subjects SLC091 and SLC92. ACMG, variant classification according to the American College of Medical Genetics and Genomics (ACMG) classification guidelines (Oza et al. 2018); UV4, likely pathogenic; UV5, pathogenic.

Table S2: Details of applied next generation sequencing methods

Case	Sequencing method	Platform	% Reads coverage ≥ 20x	Mean coverage (x reads)
SLC002	WGS	BGISeq500	88.14	36
SLC003	MIPS	NextSeq500	94.78	920
SLC012	WGS	BGISeq500	88.45	37
SLC013	MIPS	NextSeq500	91.78	900
SLC014	MIPS	NextSeq500	92.28	815
SLC015	WES	Illumina HiSeq2000	96.84	115
SLC017	WES	Illumina HiSeq2000	96.70	125
SLC018	WGS	BGISeq500	88.77	39
SLC031	MIPS	NextSeq500	93.31	517
SLC032	WGS	BGISeq500	89.62	43
SLC036	WGS	BGISeq500	89.21	41
SLC039	MIPS	NextSeq500	93.33	676
	WGS	BGISeq500	89.21	41
SLC040	WES	Illumina HiSeq4000	93.50	136
SLC043	WES	Illumina HiSeq2000	94.85	111
SLC045	MIPS	NextSeq500	92.28	590
	WGS	BGISeq500	83.83	30
SLC048	WGS	BGISeq500	88.82	38
SLC052	WES	Illumina HiSeq2000	93.77	103
SLC056	MIPS	NextSeq500	95.21	901
SLC069	WES	Illumina HiSeq2000	96.62	130
SLC070	WES	Illumina HiSeq4000	97.18	118
SLC071	WES	Illumina HiSeq4000	97.33	121
SLC073	MIPS	NextSeq500	94.99	880
SLC078	MIPS	NextSeq500	95.63	1017
SLC079	WES	Illumina HiSeq4000	97.17	101
	LRS	Sequel II PacBio	NA	12
SLC080	WES	Illumina HiSeq4000	97.40	115
	WGS	BGISeq500	85.27	30
SLC084	WES	Illumina HiSeq4000	98.01	123
SLC085	WGS	BGISeq500	80.33	30
SLC086	WES	Illumina HiSeq4000	97.34	123

WES, whole exome sequencing; WGS, short-read whole genome sequencing; MIPS, molecular inversion probe sequencing; LRS, long-read whole genome sequencing; NA, not applicable.

Table S3: Genes analyzed by MIP sequencing

ACTG1	*EPS8*	*LRTOMT*	*RIPOR2*
ADCY1	*ESPN*	*MARVELD2*	*S1PR2*
ADGRV1	*ESRRB*	*MCM2*	*SERPINB6*
AIFM1	*EYA1*	*MIR96*	*SIX1*
ATP1A2	*EYA4*	*MITF*	*SIX5*
BDP1	*GIPC3*	*MPZL2*	*SLC9A1*
BSND	*GJB2*	*MSRB3*	*SLC17A8*
CABP2	*GJB3*	*MYH14*	*SLC22A4*
CCDC50	*GJB6*	*MYH9*	*SLC26A4*
CD164	*GPSM2*	*MYO15A*	*SLC26A5*
CDH23	*GRHL2*	*MYO3A*	*SMPX*
CEACAM16	*GRM7*	*MYO6*	*SNAI2*
CIB2	*GRM8*	*MYO7A*	*SOX10*
CLDN14	*GRXCR1*	*NARS2*	*STRC*
CLIC5	*GRXCR2*	*NAT2*	*SYNE4*
CLPP	*GSDME*	*OSBPL2*	*TBC1D24*
CLRN1	*HARS2*	*OTOA*	*TECTA*
COCH	*HGF*	*OTOF*	*TJP2*
COL11A2	*HOMER2*	*OTOG*	*TMC1*
COL4A6	*HSD17B4*	*OTOGL*	*TMEM132E*
CRYM	*ILDR1*	*P2RX2*	*TMIE*
DCDC2	*KARS*	*PAX3*	*TMPRSS3*
DFNB31	*KCNE1*	*PCDH15*	*TNC*
DFNB59	*KCNJ10*	*PDZD7*	*TPRN*
DIABLO	*KCNQ1*	*PNPT1*	*TRIOBP*
DIAPH1	*KCNQ4*	*POU3F4*	*TSPEAR*
DSPP	*KITLG*	*POU4F3*	*USH1C*
EDN3	*LARS2*	*PRPS1*	*USH1G*
EDNRB	*LHFPL5*	*PTPRQ*	*USH2A*
ELMOD3	*LOXHD1*	*RDX*	*WFS1*

Table S4: Compound heterozygous or homozygous variants in arHL-associated genes

Case	Class	Gene	Transcript	cDNA	Protein	In-house AF (%)	gnomAD AF (%)	CADD_ PHRED	SIFT	PPH2	MutationTaster	SpliceAI	ACMG
SLC012	M1	*OTOGL*	NM_173591.3	c.890C>T	p.(Pro297Leu)	0.09	0.12	22.5	0	1.0	Disease causing	-	UV2
		OTOGL	NM_173591.3	c.1369G>T	p.(Val457Leu)	0.02	0.00	15.4	0	0.683	Disease causing	-	UV3

Homozygous or compound heterozygous variants detected in coding or splice site regions (+/14 nucleotides) of genes associated with autosomal recessive hearing loss (arHL). Variants are selected based on an allele frequency of ≤0.5% in gnomAD and the in-house database. Scores that meet the thresholds for pathogenicity as described in the methods section are indicated in red. Thresholds for pathogenicity: CADD-PHRED (≥15), SIFT (≤0.05), PolyPhen-2 (≥0.450), MutationTaster (deleterious) and spliceAI (≥0.1). In-house AF, allele frequency (%) in in-house database (~7,500 exomes); GnomAD AF, allele frequency (%) in gnomAD database V.2.1.1; CADD_PHRED, Combined Annotation Dependent Depletion PHRED score; SIFT, Scale-Invariant Feature Transform; PPH2, Poly-Phen-2 score; MutationTaster (prob), MutationTaster score with probability (0-1); spliceAI, splicing prediction score; ACMG, variant classification according to the American College of Medical Genetics and Genomics (ACMG) classification guidelines (Oza et al. 2018); UV2, likely benign; UV3, uncertain significance.

Table S5: List of *cis* regulatory elements of *SLC26A4*

Gene	Start	End	Source
SLC26A4, SLC26A4-AS1	106740447	106742845	GeneHancer V5.0
SLC26A4, SLC26A4-AS1	106743446	106747050	GeneHancer V5.0
SLC26A4, SLC26A4-AS1	106762501	106763480	GeneHancer V5.0
SLC26A4, SLC26A4-AS1	107103661	107105444	GeneHancer V5.0
SLC26A4, SLC26A4-AS1	107120646	107123445	GeneHancer V5.0
SLC26A4, SLC26A4-AS1	107199656	107223646	GeneHancer V5.0
SLC26A4, SLC26A4-AS1	107219645	107223646	GeneHancer V5.0
SLC26A4, SLC26A4-AS1	107232401	107238444	GeneHancer V5.0
SLC26A4-AS1	107234760	107236310	EnhancerAtlas 2.0
SLC26A4, SLC26A4-AS1	107254046	107255844	GeneHancer V5.0
SLC26A4, SLC26A4-AS1	107262447	107263690	GeneHancer V5.0
SLC26A4, SLC26A4-AS1	107276447	107280445	GeneHancer V5.0
SLC26A4, SLC26A4-AS1	107301300	107302040	EnhancerAtlas 2.0
SLC26A4, SLC26A4-AS1	107301445	107302845	GeneHancer V5.0
SLC26A4, SLC26A4-AS1	107330247	107335644	GeneHancer V5.0
SLC26A4	107334930	107335060	EnhancerAtlas 2.0
SLC26A4	107336480	107338480	EnhancerAtlas 2.0
SLC26A4	107350640	107352980	EnhancerAtlas 2.0
SLC26A4, SLC26A4-AS1	107382558	107387330	GeneHancer V5.0
SLC26A4, SLC26A4-AS1	107495047	107499844	GeneHancer V5.0
SLC26A4, SLC26A4-AS1	107531740	107533640	EnhancerAtlas 2.0
SLC26A4	107564530	107564670	EnhancerAtlas 2.0
SLC26A4, SLC26A4-AS1	107643420	107643550	EnhancerAtlas 2.0
SLC26A4	107743680	107744940	EnhancerAtlas 2.0

List of human *cis* regulatory elements associated with *SLC26A4* or *SLC26A4-AS1* that are collected in the GeneHancer database V5.0(Fishilevich et al. 2017) or the EnhancerAtlas 2.0.(Gao and Qian 2020) Only *cis* regulatory elements with an enhancer score >0.7 and an enhancer-gene interaction score >7 were extracted from Genehancer. For EnhancerAtlas 2.0, all enhancer elements that were experimentally determined in human tissues or cell types were selected. Start and End; Genomic positions on chromosome 7 according to GRCh37/hg19.

Table S6: Heterozygous variants in (predicted) *cis* regulatory elements of *SLC26A4*

Case	Class	Variant	gnomAD AF (%)	Regulatory element	Source	Identifier	Enhancer score	Enhancer-gene score	PhyloP
SLC002	M1	Chr7:107220628C>A	-1	Chr7: 107219645-107223646	GeneHancer V5	GH07J107579	2.05	10.54	-1.143
SLC045	M1	Chr7:107384987C>G	0.19	Chr7:107382558-107387330	GeneHancer V5	GH07J107742	2.25	10.63	0.183

A list of potential *cis* regulatory elements of *SLC26A4* (GeneHancer V5(Fishilevich et al. 2017) and EnhancerAtlas V2(Gao and Qian 2020)) was screened for the presence of rare heterozygous variants (allele frequency ≤0.5%) in available whole genome sequencing datasets. For none of the variants, the loss of a transcription factor binding site (TFBS) is predicted (JASPAR database(Fornes et al. 2020), >80% TFBS confidence score and a delta score of >10%). gnomAD AF, allele frequency (%) in gnomAD database V.2.1.1; Regulatory element, genomic position of regulatory element according to GRCh37/hg19; Identifier; unique identifier of regulatory element as accessible in GeneCards(Stelzer et al. 2016), Enhancer score and Enhancer-gene score of regulatory element as provided by the GeneHancer database; PhyloP(Pollard et al. 2010), nucleotide evolutionary conservation score.

Table S7: Rare genetic variants located within the CEVA haplotype

Genome	RefSNP	Location	gnomAD AF (%)	SpliceAI	PhyloP	Repeatmasker	Regulatory element
Chr7:106622156T>A	rs6961007	Intergenic	-	NA	-5.094	SINE	-
Chr7:106669858G>A (**SNP1**)	rs17424561	Intergenic	3.04	NA	-1.806	-	-
Chr7:106690778CTTTT>T	NA	Intronic (*PPKAR2B*)	-	0.01	0.556	-	-
Chr7:106736863C>T	rs149440050	Intronic (*PPKAR2B*)	3.07	0.01	0.135	LINE	-
Chr7:106741374T>C (**SNP2**)	rs79579403	Intronic (*PPKAR2B*)	3.03	0.00	0.8	LINE	GeneHancer
Chr7:106741580ATT>A	NA	Intronic (*PPKAR2B*)	-	0.01	0	LINE	GeneHancer
Chr7:106764419T>A (**SNP3**)	rs17425867	Intronic (*PPKAR2B*)	3.05	0.00	0.852	-	-
Chr7:106807591TAAAA>T	NA	Intergenic	-	NA	0.621	-	-
Chr7:106812322A>AA	NA	Intronic (*HBP1*)	-	0.00	-2.377	SINE	-
Chr7:106815154T>C (**SNP4**)	rs117113959	Intronic (*HBP1*)	2.93	0.05	-0.481	-	-
Chr7:106837681G>A (**SNP5**)	rs17349280	Intronic (*HBP1*)	2.90	0.01	0.275	-	-
Chr7:106930234C>T (**SNP6**)	rs117386523	Intronic (*COG5*)	2.92	0.00	0.641	-	-
Chr7:106967931A>G (**SNP7**)	rs80149210	Intronic (*COG5*)	2.91	0.00	0.838	LINE	-
Chr7:106993159AT>A (**SNP8**)	rs199667576	Intronic (*COG5*)	2.96	0.00	-100	-	-
Chr7:107014419A>G (**SNP9**)	rs9649298	Intronic (*COG5*)	2.90	0.00	2.769	-	-
Chr7:107081658G>A	rs188905420	Intronic (*COG5*)	2.31	0.00	-2.019	SINE	-
Chr7:107147622T>C (**SNP10**)	rs117714350	Intronic (*COG5*)	2.32	0.00	0.238	LINE	-
Chr7:107242636CT>C (**SNP11**)	rs199915614	Intronic (*BCAP29*)	1.91	NA	-100	-	-
Chr7:107282469A>C (**SNP12**)	rs150942317	Intergenic	2.31	NA	0.241	LTR	-
Chr7:107316164G>A	rs185507318	Intronic (*SLC26A4*)	2.01	0.00	0.089	SINE	-

Rare genetic variants (allele frequency ≤5% gnomAD) that are shared between two individuals that harbor the CEVA allele. SNPs that are part of the previously described CEVA (SNP 1-12) or the V1-CEVA haplotype (SNP 4-12) are underlined. Genome, Genomic position according to GRCh37/hg19; RefSNP, dbSNP reference SNP number; GnomAD AF, allele frequency (%) in gnomAD database V.2.1.1; spliceAI, highest splicing prediction score; RepeatMasker, the interspersed repeats or low complexity DNA sequence at the genomic position according to RepeatMasker; Regulatory element, overlap of the genomic position with a predicted *cis* regulatory element according to GeneHancer V5 or EnhancerAtlas 2.0; NA, not available.

Table S8: Analyzed ears of affected individuals

Class	Number of subjects (male/female)	Number of EVA ears (male/female)	Number of analyzed ears (male/female)	Average age of subjects and analyzed ears (years)	Average age of analyzed ears (Chao et al. 2019) (years), amount of analyzed ears between brackets
M2	11 (5/6)	22 (10/12)	21 (10/11)	13.2	18.4 (n = 48)
M1/CEVA	10 (6/4)	17 (10/7)	16 (9/7)	12.8	7.5 (n = 20)
M1	4 (1/3)	8 (2/6)	8 (2/6)	25 (7, 16, 18 and 59)	15.8 (n = 5)
M0/CEVA	2 (0/2)	3 (0/4)	3 (0/4)	15 (6 and 24)	10.1 (n = 6)
M0	10 (5/5)	17 (9/8)	17 (9/8)	14.6	12.9 (n = 94)

Table adapted from Chao et al. 2019 (Chao et al. 2019). Only ears with sufficient audiometric data were used in the analysis.

SUPPLEMENTARY FIGURES

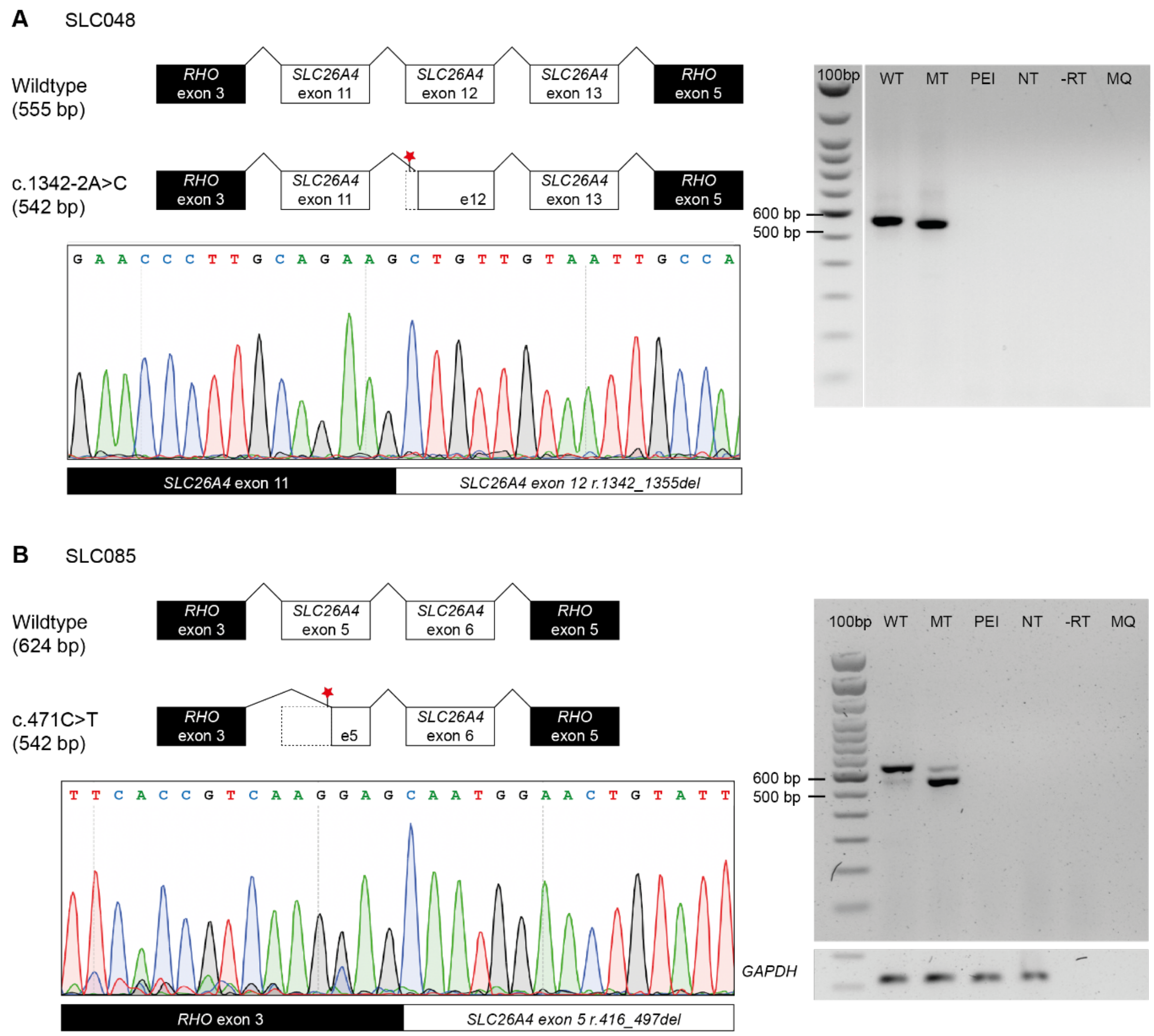

Figure S1: Results of *in vitro* splice assays for variants in SLC048 and SLC085. *In vitro* splice assays were performed in HEK293T cells to validate predicted splice defects. **(A)** In SLC048, a canonical splice site *SLC26A4* variant (c.1342-2A>C) was detected. According to SpliceAI predictions, the splice variant (MT) weakens the canonical splice acceptor site. Splice assay results revealed usage of an alternative splice acceptor site located 13 nucleotides downstream. This leads to the formation of a truncated out-of-frame exon 12 (NM_000441.1:r.1342_1355del; p.Ser448Leufs*3). **(B)** In SLC085, a synonymous variant was detected (c.471C>T, p.(Pro157=)). According to SpliceAI, the variant (MT) potentially strengthens an alternative splice acceptor site. The *in vitro* splice assay confirmed that the alternative splice acceptor site (located 27 nucleotides downstream of the variant) is used, which leads to partial exon 5 skipping (NM_000441.1:r.416_497del; p.Gly139Alafs*6,=). Bp, base pair; wt, wildtype; mt, mutant; PEI, transfection reagent-only; RT, reverse transcriptase control; MQ, water control.

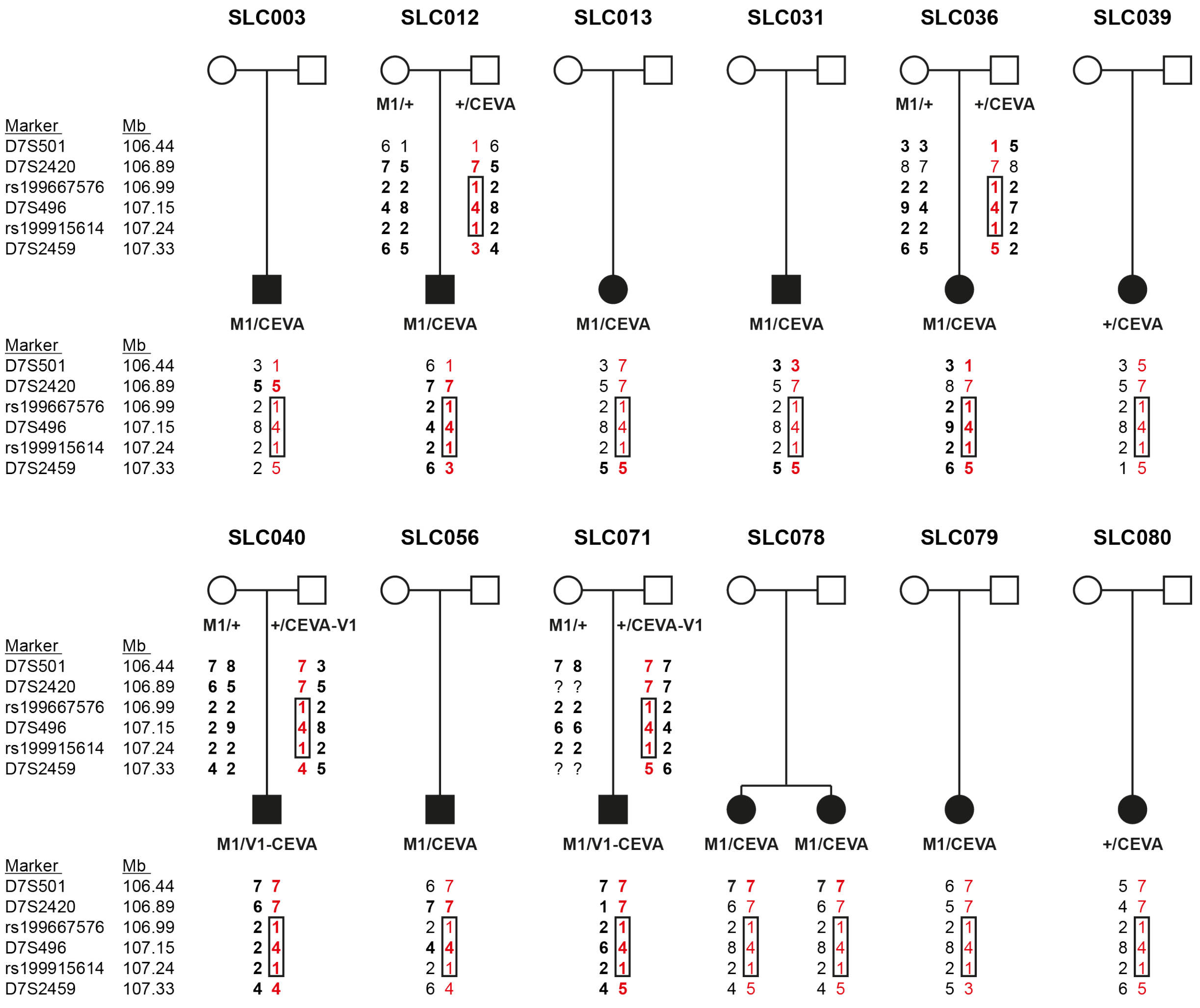

Figure S2: Family pedigrees with haplotypes of VNTR markers. The allele carrying the CEVA haplotype is marked in red. VNTR markers for which the phase of the alleles could be conclusively determined via segregation in the family are marked in bold. Genomic positions (Mb) on chromosome 7 are according to the UCSC genome browser (GRCh37/hg19). VNTR makers of the CEVA haplotype are marked in red. A shared haplotype of 0.89 Mb delimited by the markers D7S501 and D7S2459 was identified. A different repeat length was determined for marker D7S2420 in individual SLC003, the marker is still considered to be potentially part of the shared haplotype as a change of repeat length cannot be excluded. +, wildtype allele; M1, (likely) pathogenic *SLC26A4* variant.

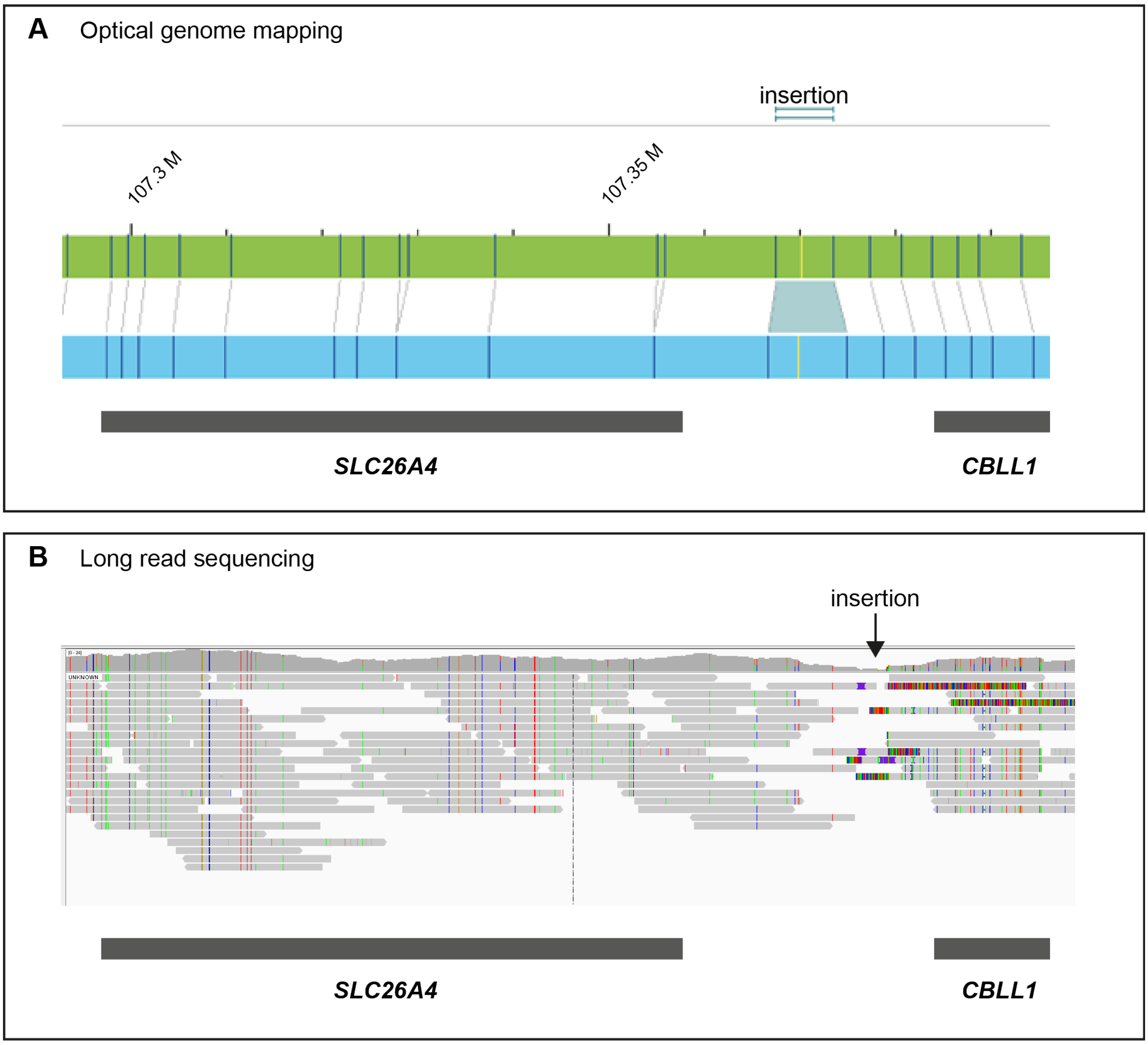

Figure S3: Optical genome mapping and long-read sequencing. Optical genome mapping (Bionano Genomics) and long-read sequencing (PacBio) were performed to detect potential structural variants (SVs) that could be present on the CEVA allele. **(A)** Optical genome mapping was performed using ultra-high molecular weight DNA isolated from peripheral blood cells from individual SLC012 (M1/CEVA). No SVs within the CEVA region or *SLC26A4* were called. One insertion event was called just telomeric from the CEVA-haplotype, but was also called in in 100% of the control samples. **(B)** Hi-Fi sequencing reads visualized in IGV software. PacBio long-read sequencing was performed on genomic DNA isolated from peripheral blood from individual SLC079 (M1/CEVA). After sequencing analyses, no SVs remained that were present within the CEVA region or *SLC26A4*. The same insertion event as depicted in A was detected using PacBio sequencing, but was also present in control data. The insertion event is therefore considered a reference problem and not a true SV.

M0

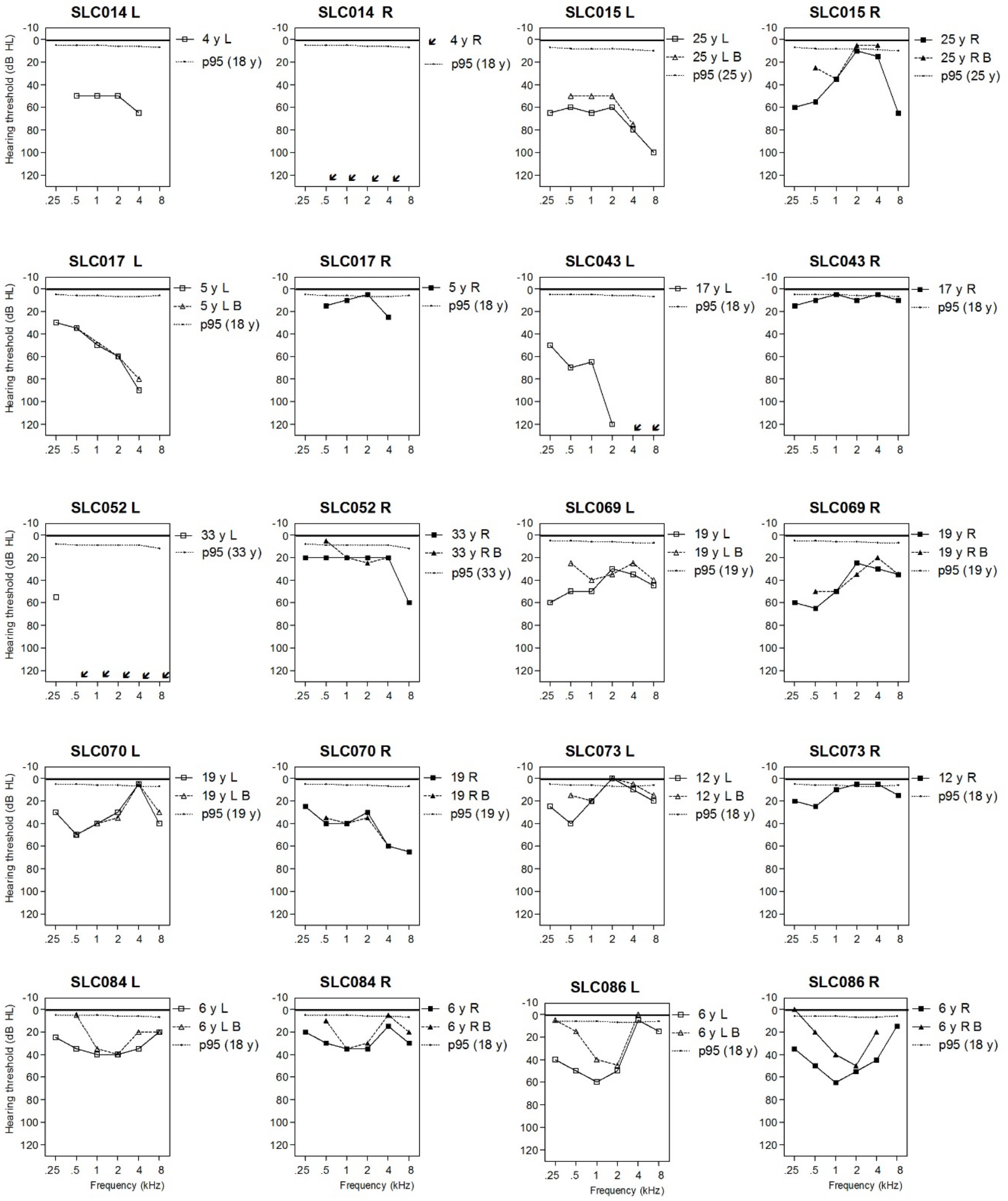

Figure continues on next page

M0/CEVA

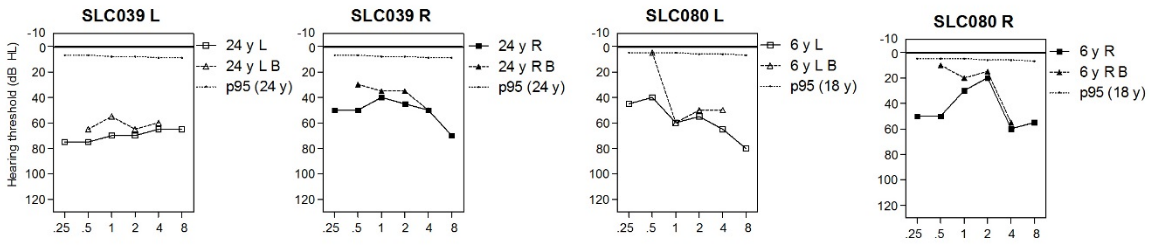

M1

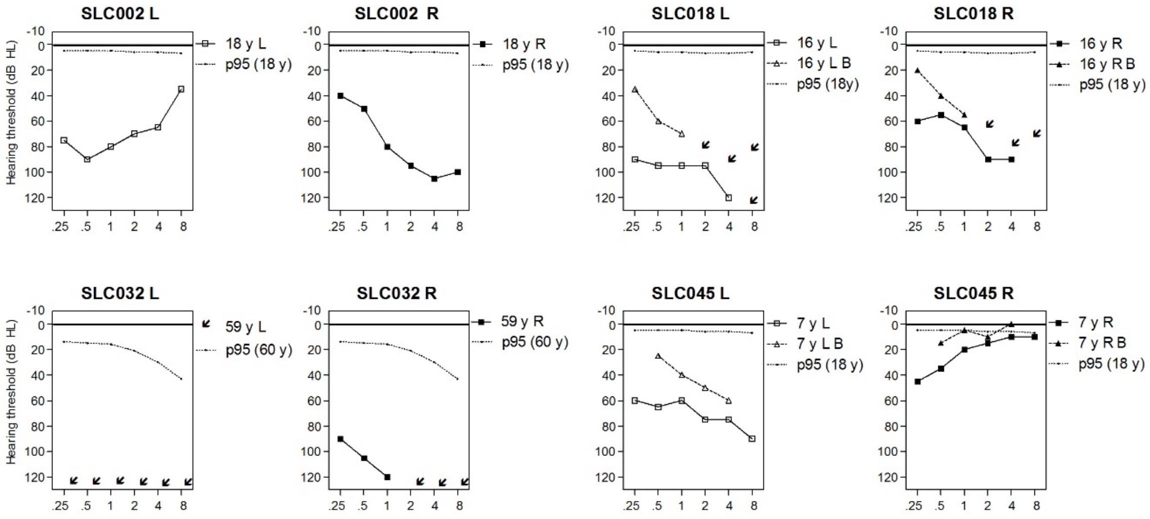

M1/(V1-)CEVA

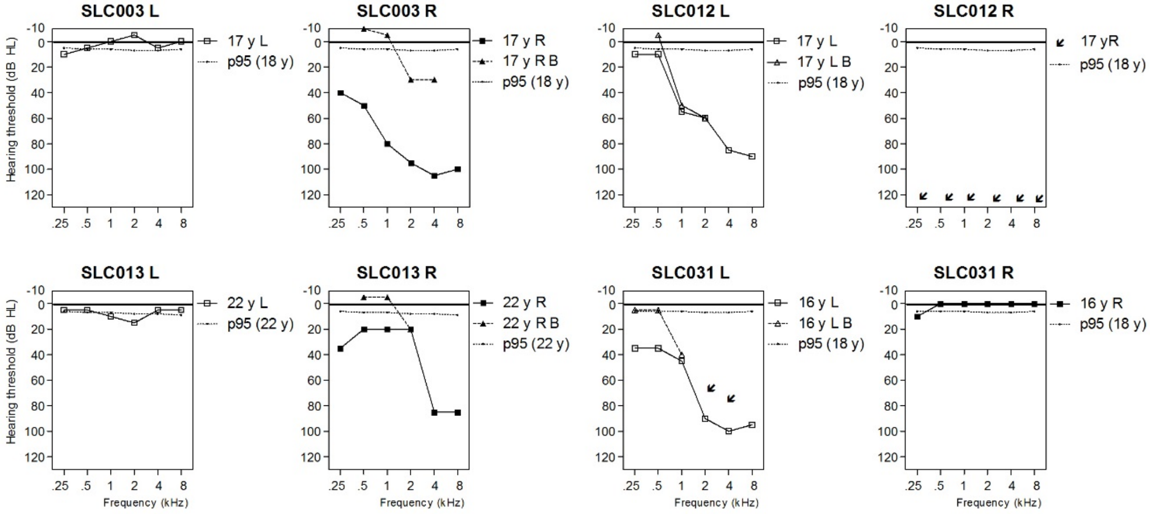

Figure continues on next page

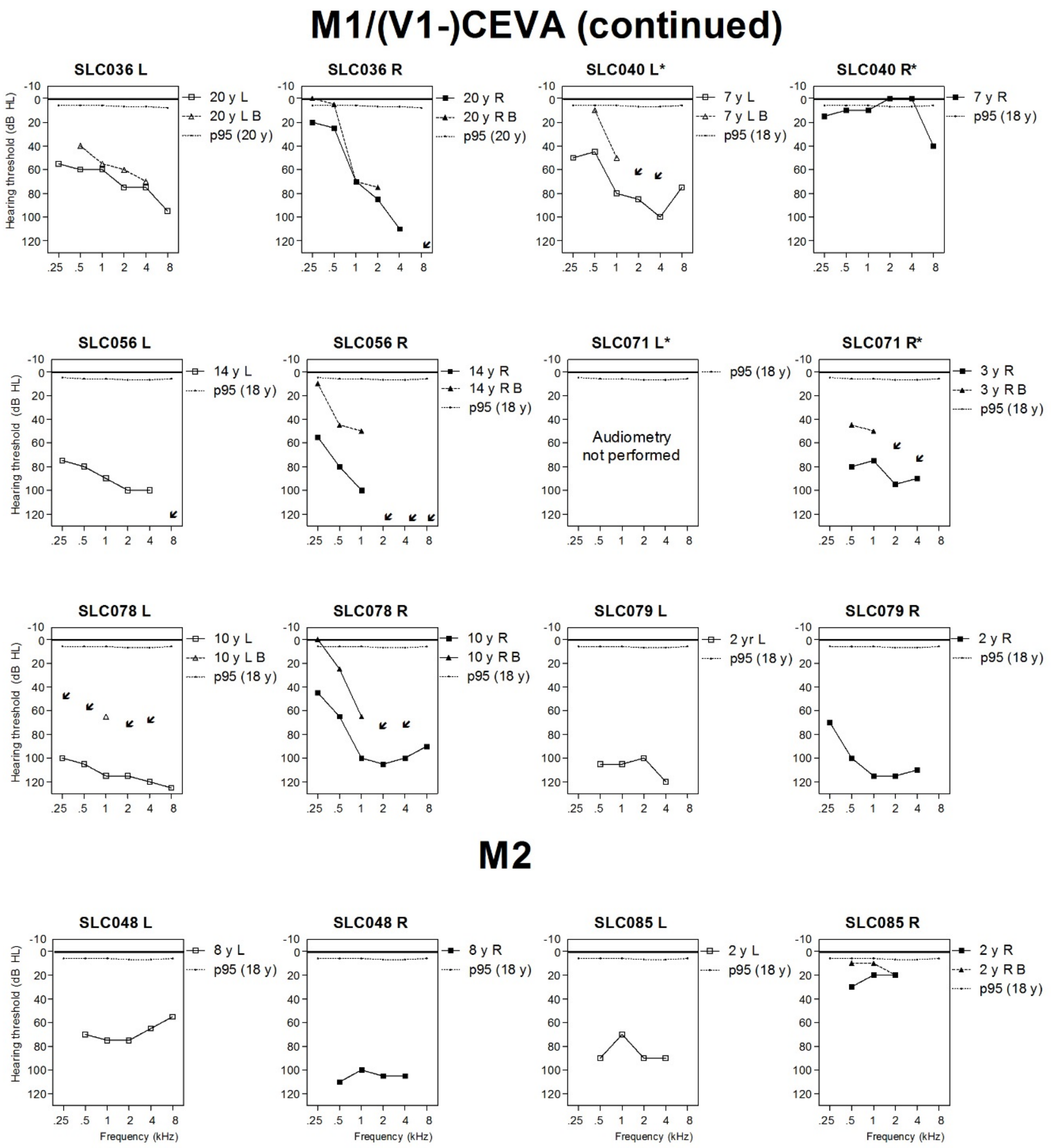

Figure S4: Pure tone audiometry of affected individuals Air, and if available, bone conduction thresholds of all subjects are depicted, except for subjects of the M2 reference cohort. The p95 values are matched to the individuals' sex and age at the most recent audiometry, according to the ISO 7029:2017 standard. The age range for which the ISO 7029:2017 can be applied is 18 to 70 years. Black arrows: threshold could not be measured. The CEVA haplotype was detected in 8 individuals, in an additional 2 individuals (SLC040 and SLC071, indicated with *), a smaller haplotype was found, termed V1-CEVA. y, age in years; R, right; L, left; B, bone conduction; dB HL, decibel hearing level; kHz, kiloHertz.

SUPPLEMENTARY REFERENCES

Chao JR, Chattaraj P, Munjal T, Honda K, King KA, Zalewski CK, Chien WW, Brewer CC, Griffith AJ (2019) SLC26A4-linked CEVA haplotype correlates with phenotype in patients with enlargement of the vestibular aqueduct. BMC Medical Genetics 20: 118. doi: 10.1186/s12881-019-0853-4

Fishilevich S, Nudel R, Rappaport N, Hadar R, Plaschkes I, Iny Stein T, Rosen N, Kohn A, Twik M, Safran M, Lancet D, Cohen D (2017) GeneHancer: genome-wide integration of enhancers and target genes in GeneCards. Database (Oxford) 2017: bax028. doi: 10.1093/database/bax028

Fornes O, Castro-Mondragon JA, Khan A, van der Lee R, Zhang X, Richmond PA, Modi BP, Correard S, Gheorghe M, Baranašić D, Santana-Garcia W, Tan G, Chèneby J, Ballester B, Parcy F, Sandelin A, Lenhard B, Wasserman WW, Mathelier A (2020) JASPAR 2020: update of the open-access database of transcription factor binding profiles. Nucleic Acids Research 48: d87-d92. doi: 10.1093/nar/gkz1001

Gao T, Qian J (2020) EnhancerAtlas 2.0: an updated resource with enhancer annotation in 586 tissue/cell types across nine species. Nucleic Acids Research 48: d58-d64. doi: 10.1093/nar/gkz980

Oza AM, DiStefano MT, Hemphill SE, Cushman BJ, Grant AR, Siegert RK, Shen J, Chapin A, Boczek NJ, Schimmenti LA, Murry JB, Hasadsri L, Nara K, Kenna M, Booth KT, Azaiez H, Griffith A, Avraham KB, Kremer H, Rehm HL, Amr SS, Abou Tayoun AN (2018) Expert specification of the ACMG/AMP variant interpretation guidelines for genetic hearing loss. Hum Mutat 39: 1593-1613. doi: 10.1002/humu.23630

Pollard KS, Hubisz MJ, Rosenbloom KR, Siepel A (2010) Detection of nonneutral substitution rates on mammalian phylogenies. Genome Research 20: 110-121. doi: 10.1101/gr.097857.109

Stelzer G, Rosen N, Plaschkes I, Zimmerman S, Twik M, Fishilevich S, Stein TI, Nudel R, Lieder I, Mazor Y, Kaplan S, Dahary D, Warshawsky D, Guan-Golan Y, Kohn A, Rappaport N, Safran M, Lancet D (2016) The GeneCards suite: from gene data mining to disease genome sequence analyses. Current Protocols in Bioinformatics 54: 1.30.1-1.30.33. doi: https://doi.org/10.1002/cpbi.5
